# Contextualizing the privacy paradox—a risk–benefit analysis of generation z’s adoption intentions toward AI-based virtual try-on

**DOI:** 10.3389/fpsyg.2026.1773754

**Published:** 2026-04-10

**Authors:** Keren Mao, Rongrong Cui, Zhicheng Wang

**Affiliations:** 1 Art and Design Academy, Zhejiang Sci-Tech University, Hangzhou, China; 2 School of Fashion Design and Engineering, Zhejiang Sci-Tech University, Hangzhou, China; 3 Digital Intelligence Style and Creative Design Research Center, Key Research Center of Philosophy and Social Sciences, Zhejiang Sci-Tech University, Hangzhou, China

**Keywords:** AI-driven virtual try-on, fsQCA, PLS-SEM, privacy calculus model, privacy paradox, theory of planned behavior

## Abstract

**Introduction:**

With the rapid advancement of artificial intelligence (AI), AI-driven virtual try-on (AI-VTO) services are reshaping consumption patterns in fashion retail. At the same time, their reliance on sensitive personal data has intensified privacy-related concerns. As digital natives and a key consumer segment, Generation Z often exhibits a “privacy paradox” in AI-enabled contexts, expressing concern about privacy while continuing to use data-intensive services. To explain this phenomenon, this study integrates the Theory of Planned Behavior (TPB) and the Privacy Calculus Model (PCM) into a unified risk–benefit framework.

**Methods:**

Survey data were collected from 709 Generation Z consumers in the Yangtze River Delta region of China. The data were analyzed using Partial Least Squares Structural Equation Modeling (PLS-SEM) and Fuzzy-set Qualitative Comparative Analysis (fsQCA).

**Results:**

The results show that perceived responsiveness, attitude, and perceived behavioral control positively influence intention to use AI-VTO services, whereas intrusiveness concerns exert a significant negative effect. In contrast, traditional privacy concerns do not have a direct effect on usage intention. Attitude mediates the effects of both perceived benefits and perceived risks on behavioral intention. In addition, the fsQCA results identify three distinct pathways leading to high adoption intention: an efficacy trust–driven pathway, an experience-driven pathway, and a control convenience–driven pathway. These findings suggest that the privacy paradox is more likely to emerge in experience-oriented contexts.

**Discussion:**

This study clarifies how Generation Z evaluates data-intensive AI services by revealing both net effects and configurational pathways underlying AI-VTO adoption. It extends current understanding of the privacy paradox in AI-enabled consumption and offers practical implications for developing transparent, user-centered, and trustworthy AI-VTO systems.

## 1 Introduction

With the rapid advancement of artificial intelligence (AI), the consumer landscape and service models of the fashion industry are being profoundly reshaped, particularly in e-commerce and digital retail. As an emerging intelligent retail technology, AI-driven virtual try-on (AI-VTO) (Krishnapriya et al., 2025) integrates image recognition, body modeling, and personalized recommendation systems to offer consumers a more intuitive, convenient, and immersive online try-on experience (Aakash et al., 2024). Compared with conventional online shopping methods, AI-VTO can reduce consumer uncertainty regarding garment fit, style compatibility, and purchase outcomes, thereby enhancing shopping efficiency and improving the overall decision-making process (Poushneh, 2018; Imark Infotech, 2024). Meanwhile, as technology companies such as Google continue to expand the application of AI try-on functions across search platforms, e-commerce ecosystems, and international markets, AI-VTO is becoming an increasingly important development direction in digital fashion retail.

However, although AI-VTO enhances the consumer experience, it also significantly increases users’ awareness that their personal data are being collected, analyzed, and utilized (Gao and Liang, 2025). Compared with conventional digital services, AI-VTO systems rely heavily on users uploading and authorizing more sensitive types of data, such as body measurements, facial images, physical characteristics, clothing preferences, and consumption behaviors (Protection, F. D, 2018; Calzada, 2022). This means that while consumers benefit from personalized, convenient, and interactive experiences, they must also bear the potential risks of privacy breaches, data misuse, and algorithmic bias (Amil, 2024; Chen and Liu, 2015). Existing research indicates that, in data-driven digital service environments, users’ trust in platforms, perceptions of risk, and evaluations of data-use transparency significantly influence their willingness to adopt and continue using such technologies (Tjahjana et al., 2024; El-Annan and Hassoun, 2025). Consequently, understanding consumers’ decision-making logic in contexts where high convenience coexists with elevated privacy risks has become a critical issue in AI retail research.

Against this backdrop, Generation Z represents a particularly important population for investigation (Negroponte, 1995). Having grown up with digital media, social platforms, and smart devices, this cohort is widely regarded as a core consumer segment characterized by high receptivity to new technologies and a strong demand for personalized and interactive shopping experiences (Priporas et al., 2017). Existing research indicates that Generation Z demonstrates high levels of technological engagement across digital environments such as e-commerce, social media, and smart retail, and is more willing to exchange personal data for convenience, entertainment, and personalized services (Vieira et al., 2020; Chitra et al., 2024). However, this tendency to embrace technology does not imply a lack of sensitivity to Privacy Concerns (PCs). On the contrary, Generation Z frequently discloses personal information while simultaneously maintaining heightened awareness of data breaches, platform surveillance, and information misuse. This coexistence of high levels of disclosure and concern exemplifies the core characteristics of the privacy paradox (Ha et al., 2024; Alić and Sopić, 2023). Consequently, Generation Z’s privacy-related decision-making in AI-VTO contexts is particularly representative and worthy of scholarly attention.

Although prior research has examined individuals’ information disclosure behavior, Privacy Concerns, and their relationship with technology adoption in contexts such as online healthcare (Sun et al., 2022), mobile internet, e-commerce (Olivero and Lunt, 2004), and intelligent services (Gao et al., 2023), and has also explained users’ decision-making mechanisms in digital environments from perspectives such as privacy calculus (Fernandes and Pereira, 2021), trust formation (Lappeman et al., 2022), and technology acceptance (Amin et al., 2024), research on AI-driven virtual try-on (AI-VTO) as a highly data-intensive context remains limited. In particular, the existing literature still exhibits several notable gaps. First, research on AI-VTO has primarily focused on technical implementation, interactive experience, or marketing effectiveness, with insufficient attention to the underlying privacy trade-off mechanisms (Kim et al., 2019). Second, although research on the privacy paradox is relatively abundant, the majority of the studies focus on general digital platforms or traditional e-commerce contexts and lack in-depth explanations of Generation Z’s cognitive and behavioral pathways in AI-driven retail settings (Kokolakis, 2017). Third, existing research rarely integrates risk–benefit trade-off logic with mechanisms of behavioral intention formation and therefore remains insufficient to systematically explain the psychological mechanisms underlying Generation Z’s decisions to use AI-VTO (Wang et al., 2016).

To address the above research gaps, this study focuses on Generation Z users’ Intention to Use (IU) AI-driven virtual try-on (AI-VTO). It integrates the Privacy Calculus Model (PCM) (Zhu et al., 2021) with the Theory of Planned Behavior (TPB) (Armitage and Conner, 2001) to develop a comprehensive framework explaining privacy trade-offs and behavioral intention formation. Specifically, the study examines perceived benefits and perceived risks by incorporating Perceived Personalization (PP), Perceived Responsiveness (PR), AI convenience, Privacy Concerns, and Intrusiveness Concerns (INCs) into the analytical model. Building on TPB, the study further investigates how Generation Z weighs Privacy Concerns against technological benefits and, in turn, forms Intention to Use AI-VTO (Dinev and Hart, 2006). In doing so, this study provides a more integrated theoretical explanation of Generation Z’s privacy decision-making logic in AI retail contexts. It offers practical implications for improving platform privacy governance and strengthening consumer trust.

## 2 Literature review

### 2.1 AI-driven virtual try-on

In the fashion industry, conventional virtual try-on (VTO) systems integrate web-based platforms, virtual reality (VR), augmented reality (AR) technologies, and digital avatars to deliver virtual fitting experiences to consumers (Deldjoo et al., 2023). VR technology generates immersive computer-simulated environments that replicate real-world settings (Steuer, 1995). In contrast, AR technology overlays digital information onto the physical environment, augmenting and modifying real-world experiences to create seamless integration between virtual and physical elements (Liu et al., 2020). Traditional VTO systems primarily depend on complex modeling and physical simulation (Sah et al., 2024), leading to cumbersome operation, limited accuracy, and poor cross-platform compatibility. Consequently, these limitations lead to suboptimal user experiences and higher maintenance costs.

With the rapid advancement of artificial intelligence (AI), virtual try-on (VTO) has become a major technological innovation in the fashion e-commerce sector. AI-VTO systems leverage the integration of computer vision (Dhatrak et al., 2024), deep learning algorithms (Marelli et al., 2022), and three-dimensional (3D) human-body modeling (Roth et al., 2016) to accurately superimpose garments onto digital avatars. This integration provides consumers with an immersive and interactive online shopping experience (Batool and Mou, 2024). Beyond enhancing user engagement and purchase intention, accurate matching algorithms reduce product returns arising from size or style mismatches (Chen W. et al., 2024) and help alleviate inventory pressures for retailers (Huang and Liao, 2015).

To clarify this perceptual gap, this study conceptually compares traditional virtual try-on with AI-powered virtual try-on. Traditional virtual try-on mainly relies on digital simulation (Hilken et al., 2017) and augmented reality (AR) to present fitting effects, with an emphasis on perceptual realism, immersive interaction, and technological experience (Rauschnabel et al., 2022). By contrast, AI-powered virtual try-on is driven by artificial intelligence algorithms and characterized by stronger personalization and more intelligent interaction (Davenport et al., 2020), but it may also introduce algorithmic bias and ethical risks. This comparison identifies technological logic, data dependence, personalization, and privacy risk as the key dimensions distinguishing the two approaches. More importantly, it underscores consumers’ privacy-related concerns: AI-VTO not only requires the collection of body measurement data (Youn et al., 2023) but also depends on in-depth analysis of such data to generate personalized recommendations and predict consumer preferences. Although this data-driven process improves the try-on experience, it may also heighten concerns about privacy disclosure, because sensitive information such as body measurements, clothing habits, and shopping preferences can potentially be used for unauthorized analysis or commercial purposes (Elliott and Soifer, 2022; Nan et al., 2025).

As an increasing number of fashion brands adopt AI-VTO technologies, consumers are engaging in more personalized and immersive shopping experiences while simultaneously becoming increasingly aware of the associated privacy risks. For instance, Google Shopping’s generative AI fitting tool (Nagy and Hajdú, 2021), Taobao’s AI try-on service (Kempa et al., 2020; Lau and Ki, 2021), and Nike’s digital footwear simulation (Zaheer, 2025) have collectively transformed the online shopping experience through data-driven innovation. However, these innovations have simultaneously heightened consumer concerns about data collection practices, algorithmic transparency, and individual autonomy over personal information (Durgade et al., 2024).

Overall, consumers evaluate both perceived value and privacy risk when deciding whether to use AI-VTO technologies. However, existing studies have not reached a consensus regarding consumers’ willingness to use such technologies. On the one hand, some scholars argue that AI’s personalization and convenience enhance perceived experiential value and, in turn, strengthen usage intentions (Awad and Krishnan, 2006); on the other hand, the processing of sensitive biometric data—such as facial images and body measurements—continues to elicit considerable privacy and ethical concerns. Even when AI technologies provide substantial utilitarian and experiential benefits, consumers’ defensive psychological barriers often persist (Feng and Xie, 2019). Therefore, developing a more comprehensive theoretical framework is essential to systematically explain how consumers form their final willingness to use AI-VTO technologies within a risk–benefit trade-off mechanism.

### 2.2 Theoretical background

The Theory of Planned Behavior (TPB) offers a systematic explanation of behavioral intentions through three core constructs: Attitude (AT), subjective norm, and Perceived Behavioral Control (PBC) (Ajzen, 1991). Within the AI-VTO context, this framework can be viewed as a typical model for explaining technology adoption behavior. According to TPB, both an individual’s Attitude—defined as the positive or negative cognitive evaluation of a system—and Perceived Behavioral Control—representing self-assessed capability to manage privacy risks—jointly influence willingness to use the technology (Wei W. et al., 2025). However, recent longitudinal and meta-analytic studies have identified a substantial gap in the “privacy Attitude–disclosure behavior” pathway, commonly referred to as the privacy paradox (Kim et al., 2023). This suggests that although Attitude remains a central construct in behavioral prediction, it alone cannot fully explain temporal inconsistencies and dynamic discrepancies between Attitudes and actual behaviors (Barth and De Jong, 2017).

Nonetheless, Attitudes can function as mediating variables whose influence is moderated by contextual factors such as technological experience (Chen et al., 2025) and risk–benefit trade-offs (Meier and Krämer, 2024), thereby helping explain why actual behavior often diverges from predictions based solely on Attitude. Furthermore, incorporating Perceived Behavioral Control helps preserve the model’s structural integrity and theoretical coherence. Previous research indicates that Perceived Behavioral Control reflects users’ subjective sense of mastery over technological complexity and risk, addressing the limitations of relying solely on Attitudes to explain usage intentions. This enhances the TPB framework’s applicability and predictive validity within AI-driven virtual try-on contexts. Because AI virtual fitting typically occurs in independent, individualized online environments that lack direct social observation or evaluation, the constraining influence of subjective norms on decision-making is relatively diminished (Suler, 2004). Generation Z users, in particular, emphasize autonomy and digital self-control; their privacy-related decisions are more strongly driven by perceived risk, technological experience, and trust mechanisms than by external social expectations (Guerra-Tamez et al., 2024).

The Privacy Calculus Model (PCM) offers a theoretical framework for explaining how individuals evaluate perceived risks and benefits when deciding whether to disclose personal information (Dinev and Hart, 2006). Compared with traditional models, PCM places greater emphasis on cognitive and environmental factors that influence privacy decision-making. Its core assumption is that consumers rationally balance potential negative consequences against anticipated gains. According to PCM, privacy decision-making involves a rational calculus through which users assess both the immediate benefits and the long-term risks of AI applications prior to disclosure (Beke et al., 2022). Integrating PCM introduces a risk–benefit evaluative logic into the Attitude and Perceived Behavioral Control components of the Theory of Planned Behavior (TPB). This integration reconceptualizes Attitude from a simple affective evaluation to a deliberative judgment grounded in cognitive assessment. Simultaneously, Perceived Behavioral Control is strengthened through users’ heightened perception of control over privacy risks. This mechanism helps explain the privacy paradox, wherein users express Privacy Concerns yet still disclose personal information, as their decisions are significantly shaped by the mediating influence of anticipated benefits.

The proposed TPB–PCM integrated framework provides a more comprehensive explanation of Generation Z’s intention to adopt AI-VTO technologies. Specifically, TPB captures cognitive evaluations through the constructs of Attitude and Perceived Behavioral Control, whereas PCM highlights the risk–benefit trade-off between Privacy Concerns and perceived functional gains. Together, these models constitute a dynamic decision-making mechanism in which users form their behavioral intentions by cognitively weighing perceived risks against expected utility.

### 2.3 Theoretical perspectives on the privacy paradox

The privacy paradox refers to the phenomenon in which individuals express high levels of Privacy Concern at the attitudinal level, yet continue to disclose personal information in their actual online behaviors. Extensive research across e-commerce (Tsai et al., 2011), social networking (Taddicken, 2014), mobile applications (Keith et al., 2013), and other digital contexts has consistently validated this Attitude–behavior inconsistency, establishing it as a central issue in information privacy research.

Barth and De Jong (2017) conducted a systematic literature review that offered a structured overview of the theoretical explanations for the privacy paradox. They suggested that the existing literature can be broadly divided into two forms of decision-making logic: one based on risk–benefit trade-offs and the other characterized by limited or absent risk assessment. This typology provides a clear theoretical basis for understanding the privacy paradox. Moreover, the privacy paradox is not a fixed, immutable individual trait but rather a context-dependent behavioral pattern. Variations in interaction rhythms, reward mechanisms, transparency, and perceived control across technological environments may significantly reshape how individuals weigh risks and benefits in their decision-making processes (Acquisti et al., 2015).

Building on this foundation, it is evident that although existing research has explained the privacy paradox from multiple perspectives—including rational trade-offs, cognitive biases, and situational factors—two important limitations remain. First, the majority of the studies focus primarily on the net effects of variables, paying insufficient attention to how risks and benefits are integrated into psychological structures and subsequently transformed into behavioral intentions. Second, in generative AI and highly data-dependent interactive environments, empirical research remains limited as to whether traditional privacy calculus logic can adequately explain users’ adoption decisions, particularly about the distinct roles played by different types of risk in the formation of behavioral intention (Kumar et al., 2026).

To address the above limitations, this study defines its research objective as investigating how users form Intention to Use AI-driven virtual try-on (AI-VTO) in a highly data-dependent context through risk–benefit trade-offs, while further examining the pathways through which different risk and benefit factors shape behavioral intention formation. This objective not only responds to the need to extend privacy paradox research into AI consumption settings but also provides a direct theoretical rationale for integrating the Theory of Planned Behavior (TPB) with the Privacy Calculus Model (PCM).

### 2.4 Generation z’s intention to use AI-VTO

Generation Z is widely regarded as a cohort of digital natives who have grown up with mobile internet, social media, and smart devices. Compared with other age groups, this cohort demonstrates higher levels of engagement with digital technologies and places greater emphasis on personalized, interactive, and immersive consumption experiences. In the context of AI-driven virtual try-on (AI-VTO), these characteristics make Generation Z particularly likely to develop Intention to Use such technologies. Existing research indicates that Generation Z consumers show a strong Intention to Use online virtual try-on services, and that this intention is closely associated with perceived convenience, entertainment value, interactivity, and personalization (Qasem, 2021).

However, Generation Z’s acceptance of AI-VTO does not imply insensitivity to Privacy Concerns. On the contrary, existing research suggests that, in digital personalization services, Generation Z often demonstrates both an expectation of benefits and a heightened awareness of privacy-related costs (McKee et al., 2024). On the one hand, they are willing to leverage personal data to obtain more accurate recommendations, more convenient decision support, and consumption experiences that better reflect their needs for self-expression. On the other hand, they remain highly concerned about data tracking, excessive profiling, algorithmic opacity, and the potential misuse of personal information. This dual tendency indicates that Generation Z’s adoption of AI-VTO is not a simple process of technological acceptance, but rather a dynamic evaluative process shaped by ongoing risk–benefit trade-offs.

This characteristic is especially salient in AI-VTO contexts. Compared with general digital services, AI-VTO typically requires more sensitive data inputs, including body images, facial features, body measurements, and shopping preferences. As a result, users not only evaluate the system’s utility and attractiveness, but also consider whether data disclosure is warranted, whether platform control mechanisms are sufficient, and whether algorithmic processing is transparent. Existing research suggests that although Generation Z may not continuously attend to algorithms themselves, they are highly sensitive to issues of data control. When users perceive greater control, more intuitive privacy settings, and stronger algorithmic explainability, they are more likely to accept such services and disclose personal information. By contrast, when platforms present complex privacy policies, cumbersome settings, or ambiguous data practices, Generation Z becomes more vulnerable to privacy fatigue, which in turn reduces their willingness to manage personal information actively (Brake, 2022).

**Figure 1:**
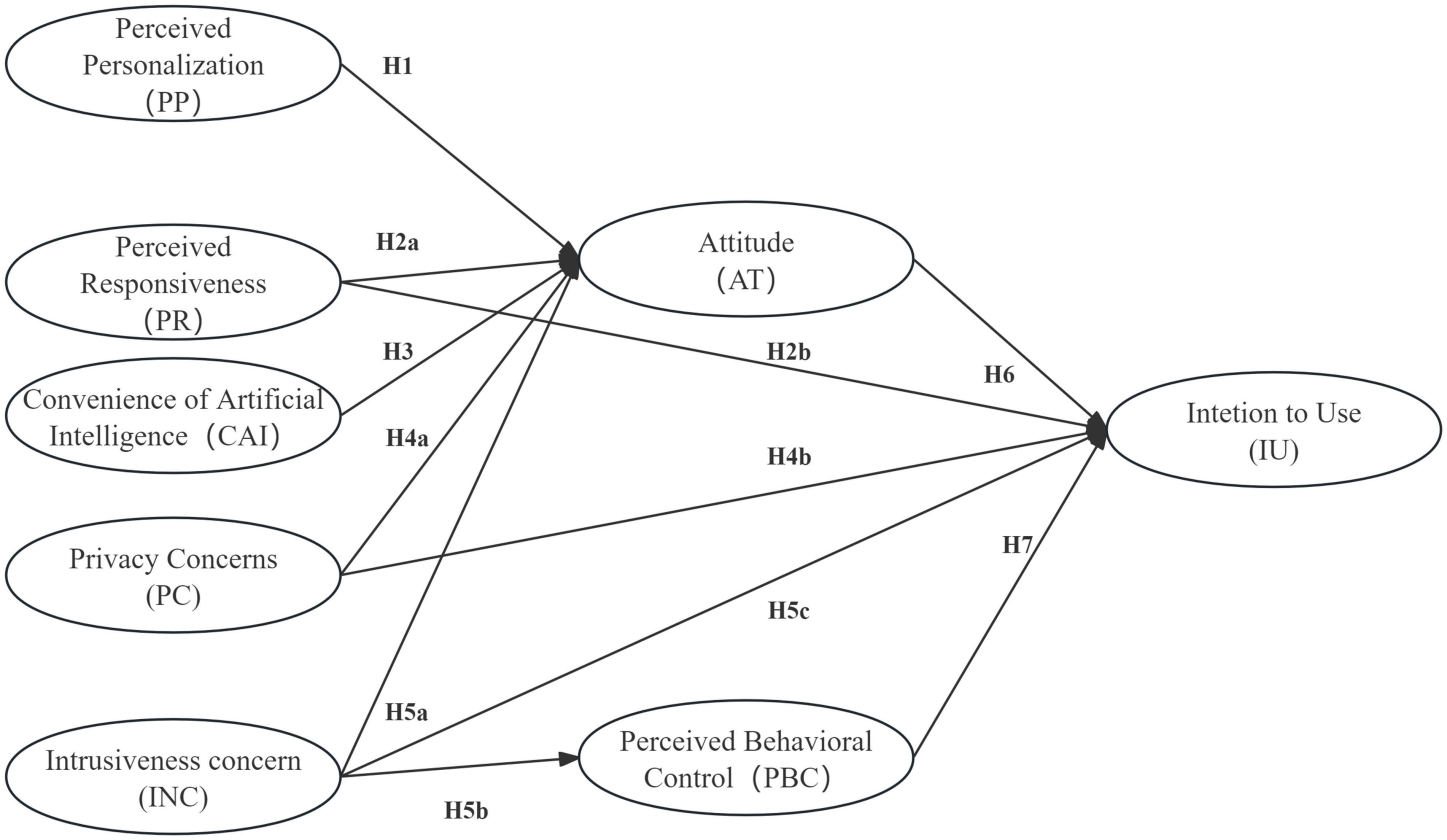
Conceptual research model based on the integrated TPB–PCM framework.

Therefore, in the context of AI-VTO adoption, Generation Z should not be characterized solely by high technological receptivity. This cohort also demonstrates strong demands for personalization, heightened sensitivity to data-related issues, and a substantial reliance on control mechanisms. Accordingly, analyses of Generation Z’s willingness to use AI-VTO should extend beyond perceived usefulness and experiential value to include evaluations of privacy risks, data control, and algorithmic transparency. On this basis, the present study identifies Generation Z as its target population. It investigates how personalized benefits, convenience, and privacy risks are balanced in AI-VTO contexts to shape usage intentions. Building on the preceding literature review, the next section develops a conceptual research model and corresponding hypotheses (see Figure 1) to examine these underlying mechanisms in greater depth.

### 2.5 Research framework and hypothesis development

#### 2.5.1 Perceived personalization

Perceived Personalization (PP) refers to the extent to which consumers perceive that AI-powered virtual try-on (VTO) systems generate customized try-on results and recommendations tailored to their body shape, facial features, and personal preferences. It fundamentally captures the degree of alignment between system outputs and individual user needs (Xu et al., 2023).

In virtual try-on contexts, Perceived Personalization represents a key external characteristic of the system. It allows users to perceive that the service aligns more closely with their individual needs and preferences, thereby enhancing perceived usefulness and ease of use (Jiang et al., 2021). In digital retail and augmented reality shopping settings, higher levels of personalization increase the likelihood that consumers will regard system-provided information as personally relevant, which in turn strengthens their perception of the technology’s usefulness and their overall evaluation of the system. In the AI-VTO context, personalization not only improves the accuracy of try-on results but also reduces uncertainty in size evaluation and style matching, thereby facilitating the formation of positive Attitudes (Chen C. et al., 2024). Although personalized services may generate concerns about data use, existing studies suggest that consumers often maintain favorable evaluations because of the convenience, relevance, and experiential value such services provide (Xu et al., 2011). Accordingly, when Generation Z consumers perceive AI-VTO systems as responding accurately to their individual needs, they are more likely to develop a positive Attitude toward the technology (Voicu et al., 2023). Therefore, the following hypothesis is proposed:

*H1*: Perceived Personalization positively influences Generation Z’s Attitude toward AI-VTO.

#### 2.5.2 Perceptual responsiveness

Perceived Responsiveness (PR) refers to the extent to which users perceive system feedback as timely, relevant, and high-quality in response to their inputs during interactions with virtual try-on services. It reflects whether the technology can respond quickly, accurately, and in a personalized manner to user needs throughout the interaction process (Jiang et al., 2021). As a key dimension of perceived interactivity, Perceived Responsiveness captures not only the timeliness of system reactions, but also the relevance and effectiveness of the feedback provided. Accordingly, it shapes whether users perceive the interaction as smooth, efficient, and trustworthy (Gefen and Straub, 2000).

Extensive research shows that high Perceived Responsiveness significantly enhances users’ sense of immersion and overall interactive experience (Youn et al., 2023). When AI-driven virtual agents provide timely, relevant, and appropriate responses, they can substantially improve the customer experience (Truong and Chen, 2025). This effect is particularly salient in AI-VTO contexts, where users depend on immediate system feedback to complete virtual try-on, comparison, and selection tasks. Systems that generate relevant and useful try-on results promptly are more likely to foster positive user Attitudes (Ngo et al., 2026). In addition, Perceived Responsiveness directly shapes willingness to adopt, because high-quality feedback reduces operational uncertainty and enhances perceptions of system efficiency and usefulness, thereby strengthening users’ intention to continue using or adopt the technology (Ngo et al., 2025). Prior research also suggests that a smooth feedback process enhances user trust and satisfaction, which in turn reinforces adoption intention and long-term willingness to use the technology (Wu et al., 2007; Pavlou and Gefen, 2004). Therefore, Perceived Responsiveness functions not only as a key determinant of initial user Attitudes but also as an important mechanism supporting the sustained adoption of virtual try-on technology. Accordingly, the following hypotheses are proposed:

*H2a*: Perceived Responsiveness positively influences Generation Z’s Attitude toward AI-VTO technology.

*H2b*: Perceived Responsiveness positively influences Generation Z’s willingness to use AI-VTO technology.

#### 2.5.3 Convenience of artificial intelligence

The Convenience of Artificial Intelligence (CAI) refers to the extent to which consumers perceive AI technology as enabling them to complete shopping-related tasks with less time, effort, and operational cost. It is typically reflected in greater efficiency, more flexible modes of use, and smoother service processes (Pillai et al., 2020).

In digital consumption contexts, convenience is widely recognized as a key determinant of user Attitudes, as streamlined services reduce decision-making burdens and enhance consumers’ positive evaluations of technological utility (Sari et al., 2022). In AI-driven shopping services, convenience typically derives from automated recommendations, rapid responses, simplified processes, and enhanced information-matching efficiency. These characteristics enhance consumers’ perceptions of service fluency and practical value (Lopes et al., 2025). In the AI-VTO context, such convenience is primarily reflected in consumers’ ability to complete fitting, comparison, and selection processes more efficiently, thereby reducing the time costs and operational burdens associated with physical try-ons and repetitive searches (Shih and Yeh, 2025). When consumers perceive AI-VTO as making the shopping process more efficient, time-saving, and easier to use, they are more likely to develop positive Attitudes toward the technology, because convenience itself constitutes an important dimension of technological value (Chekembayeva et al., 2023). Accordingly, the following hypothesis is proposed:

*H3*: Perceived Convenience of Artificial Intelligence positively influences Generation Z consumers’ Attitudes toward AI-VTO.

#### 2.5.4 Privacy concerns

Privacy Concerns (PCs) refer to consumers’ apprehensions about uncertainties and potential risks associated with AI-VTO systems during the processes of data collection, storage, and utilization (Serravalle et al., 2022). In virtual fitting contexts, such concerns may involve the misuse of facial recognition data, unauthorized disclosure of body-shape information, or improper analysis of personal shopping behavior. When consumers perceive elevated privacy risks, they are likely to adopt skeptical Attitudes toward the technology, thereby reducing their overall disposition and willingness to adopt it (Protection, F. D, 2018). In virtual fitting and 3D body-scanning scenarios, the empirical research of Rauschnabel et al. (2022) revealed that users’ intentions to adopt the technology significantly declined due to concerns about the misuse of body-image data, illustrating the inhibitory effect of Privacy Concerns on usage willingness. Similarly, Lutz and Tamó-Larrieux (2020) observed that users’ adoption intentions markedly declined when they perceived heightened privacy risks related to social robots or had experienced negative interactions with such technologies. Accordingly, this study proposes the following hypotheses:

*H4a*: Privacy Concerns negatively influence Generation Z’s Attitudes toward AI-VTO technology.

*H4b*: Privacy Concerns negatively influence Generation Z’s willingness to adopt AI-VTO technology.

#### 2.5.5 Intrusiveness concern

Intrusiveness Concern (INC) refers to the discomfort or sense of violation users experience when they perceive a technology as excessively encroaching upon their daily activities or personal boundaries (Shoukat et al., 2025). This concept differs from general privacy risks by emphasizing the psychological disturbance and negative emotional experiences that arise from technology use. Within the context of AI-VTO, such concerns often stem from users’ perceptions that platforms excessively collect or utilize sensitive data, such as facial and body imagery. Prior studies indicate that heightened Intrusiveness Concerns trigger negative emotional responses, thereby significantly weakening users’ Attitudes toward and acceptance of the technology.

Cloarec (2022) identified privacy-control mechanisms as an effective means of mitigating intrusive concerns in data-driven marketing. Specifically, when users can control how their personal information is used—such as through privacy settings or data-access permissions—their perceived intrusiveness during data processing decreases significantly. As these concerns diminish, users’ sense of self-efficacy and perceived control over the technology increase, thereby enhancing their Perceived Behavioral Control (PBC). In the evolving context of ChatGPT use, perceptions of privacy or ethical intrusion by AI have been shown to reduce users’ Perceived Behavioral Control (Polyportis, 2024). In mobile-application contexts, Park et al. demonstrated that in-app advertisements evoke negative emotional responses through varying degrees of perceived intrusiveness, with notable differences between native and non-native formats (Park et al., 2020). Less intrusive advertisements, by contrast, foster more positive Attitudes and behavioral responses (Bano et al., 2022). Furthermore, Boerman et al. (2017) found that overly personalized advertisements can be perceived as intrusive, thereby reducing click-through rates and purchase volumes. Similarly, Aguirre et al. (2016) observed that perceived privacy intrusiveness can reduce user interaction. Based on these insights, this study proposes the following hypotheses:

*H5a*: Intrusiveness Concern (INC) negatively influences Generation Z’s Attitudes toward AI-VTO technology.

*H5b*: Intrusiveness Concern (INC) negatively influences Generation Z’s Perceived Behavioral Control regarding AI-VTO technology.

*H5c*: Intrusiveness Concern (INC) negatively influences Generation Z’s willingness to adopt AI-VTO technology.

#### 2.5.6 Attitude

Attitude (AT) refers to an individual’s overall evaluation of a specific behavior, reflecting the extent to which they perceive its consequences as positive or negative (Kim et al., 2023). Attitudes are typically shaped by individuals’ beliefs about behavioral outcomes and their evaluations of the significance of these outcomes, and they constitute a core determinant of behavioral intention (Fishbein and Ajzen, 2011). In this study, Attitude is conceptualized as individuals’ overall evaluation of value dimensions—including perceived benefits and perceived risks—associated with AI-VTO technology.

The positive association between consumer Attitudes and usage intentions has been widely supported in AI-related research. Kang et al. (2024) found that acceptance Attitudes exert a significant positive effect on continued usage intentions, indicating that users with favorable Attitudes toward generative AI services are more likely to sustain their use over time. Similarly, Moon’s research revealed that users’ Attitudes toward generative AI applications not only affect their acceptance but also directly shape their usage intentions by fostering trust and emotional resonance (Moon, 2024). Accordingly, this study proposes the following hypothesis:

*H6*: Attitude positively influences Generation Z’s intention to use AI-driven virtual try-on (AI-VTO) technology.

#### 2.5.7 Perceived behavioral control

Within the Theory of Planned Behavior (TPB), Perceived Behavioral Control (PBC) refers to an individual’s subjective perception of having the necessary resources and capabilities to perform a specific behavior (Ajzen and Schmidt, 2020). In artificial intelligence contexts, this perception manifests as users’ belief in their ability to interact effectively with, and exert control over, AI systems. This construct is critical because it directly shapes users’ trust in AI technologies and their willingness to use them (Lopes et al., 2024).

Prior research indicates that when users perceive greater control over AI, their perceived risks and distrust decrease significantly. This sense of control typically arises from multiple factors, including technological familiarity, system transparency, and the flexibility to customize interactions. Essentially, perceived control serves as a bridge between users and complex AI technologies, reducing anxiety and strengthening trust (Esmaeilzadeh, 2020). In this context, users who experience a stronger sense of mastery are more likely to explore and integrate AI technologies into their daily routines actively. Enhanced perceived control not only reduces perceived risks associated with AI but also strengthens technological trust, thereby motivating users to experiment with, learn from, and effectively use AI system functionalities (Wang et al., 2023). Accordingly, this study proposes the following hypothesis:

*H7*: Perceived Behavioral Control positively influences Generation Z’s willingness to use AI-VTO technology.

## 3 Research methodology

### 3.1 Statistical techniques

Partial Least Squares Structural Equation Modeling (PLS-SEM) is a variable-oriented analytical approach used to test proposed hypotheses, and the analyses in this study were conducted using the SmartPLS software (Wong, 2013). PLS-SEM is particularly suitable for research contexts characterized by small sample sizes, non-normal data distributions, complex multivariate models, and formative measurement structures, and it has been widely applied in e-commerce research (Sharma and Aggarwal, 2019). This quantitative technique effectively evaluates the relationships among multiple independent and dependent variables, making it well-suited to the complex model structure employed in this study (Wilson, 2019). Fuzzy-set Qualitative Comparative Analysis (fsQCA) focuses on identifying configurational relationships among multiple antecedent conditions from a set-theoretic perspective, effectively complementing the limitations of the PLS-SEM approach (Kraus et al., 2018). Accordingly, this study adopts a combined PLS-SEM and fsQCA methodological approach to validate the structural relationships among factors influencing Generation Z’s AI-VTO usage intentions and to interpret the diverse configurational pathways leading to behavioral outcomes.

### 3.2 Variable definition and measurement

This study aims to examine the key factors influencing Generation Z’s willingness to disclose personal information in AI-VTO contexts in China. The questionnaire was adapted from multi-item scales previously validated in studies on technology acceptance, privacy trade-offs, and consumer behavior. It was refined to suit the specific research context of AI-VTO technology. A bilingual back-translation procedure was employed to ensure linguistic accuracy and cultural equivalence. The initial translation and back-translation were conducted by two bilingual experts specializing in information management and consumer psychology, respectively. Cross-referencing between the two versions helped eliminate semantic ambiguities, and revisions were made to ensure item clarity and content validity. A seven-point Likert scale (1 = “Strongly disagree”; 7 = “Strongly agree”) was used to measure latent variables, including Perceived Personalization, Perceived Responsiveness, Privacy Concerns, Intrusiveness Concerns, and Perceived Behavioral Control. This measurement approach captures respondents’ nuanced perceptions and the intensity of their attitudinal responses.

To ensure a consistent understanding of the AI-VTO concept, Generation Z participants were first shown a short explanatory video and a series of static demonstration images before completing the questionnaire. These materials illustrated the operational process of the AI-driven virtual fitting system, including facial and body image uploads, garment-overlay effects, and the personalized recommendation interface. After viewing the demonstrations, participants interacted with Google’s virtual try-on functionality to gain direct experiential familiarity with AI-based fitting systems. This procedure helped mitigate subjective biases stemming from cognitive differences among participants, particularly in their responses to items on privacy risk perception, perceived usefulness, and immersive experience. Furthermore, to ensure that the measurement items authentically captured Generation Z’s privacy trade-offs and behavioral intentions, this study drew upon classic scales from the Privacy Calculus Model (PCM) and the Theory of Planned Behavior (TPB), adapting their semantic phrasing to the AI-VTO context. This approach not only enhanced the contextual relevance of the scales but also strengthened construct content validity, ensuring that the collected data accurately represented Generation Z’s genuine preferences in privacy-sensitive environments.

Perceived Personalization (PP; *α* = 0.88) was measured using four items adapted from Smink et al. (2020), which examine how personalized design elements in augmented reality (AR) technologies capture user attention and influence content processing and interpretation. These items were reformulated to capture the perceived degree of customized clothing recommendations generated by the AI-VTO system based on individual body shape, style preferences, and prior usage behavior. Perceived Responsiveness (PR; *α* = 0.90) was measured using three items adapted from Youn et al. (2023), who examined the responsiveness of virtual fitting services based on 3D body-scanning technologies to consumer interaction patterns. In this study, the items were modified to evaluate the AI-VTO platform’s pace of responsiveness, feedback accuracy, and effectiveness in addressing user inputs. Convenience of AI (CAI; *α* = 0.88) was measured using four items adapted from Shelvia et al. (2020), who explored how intelligent service designs in hospitality contexts reduce users’ time and effort expenditure. In this study, CAI captures Generation Z’s perceived Convenience of AI-VTO technology. Privacy Concerns (PCs α = 0.87) were measured using three items adapted from Shoukat et al. (2025), which originally assessed consumer concerns about data usage and information protection in AI-supported social commerce platforms. These items were reformulated to evaluate Generation Z’s apprehensions regarding the AI-VTO platform’s collection, storage, and use of personal information. Intrusiveness Concerns (INCs; *α* = 0.92) were measured using three items adapted from Lin and Kim (2016), which focus on perceived disruption when online advertisements are overly personalized. In this study, the items assessed Generation Z users’ perceived intrusiveness regarding the potential overcollection or use of personal data by AI-VTO platforms. Perceived Behavioral Control (PBC; *α* = 0.86) was measured using four items adapted from Nguyen et al. (2025), reflecting Generation Z users’ perceived ability to autonomously manage their personal information and privacy settings on AI-VTO platforms. Attitude (AT; *α* = 0.87) toward AI-VTO technology was measured using four items adapted from Lin and Chuang (2018), evaluating users’ overall evaluations and affective dispositions toward AI-VTO use. Intention to Use (IU; α = 0.92) was measured using three items adapted from Ruiz-Herrera et al. (2023) that focus on Generation Z consumers’ willingness and behavioral tendency to adopt AI-VTO technology.

A pre-test was conducted with 30 fashion design students to evaluate the questionnaire’s clarity, contextual relevance, and content validity. The pre-test results indicated satisfactory internal consistency and measurement stability, with an overall Cronbach’s *α* of 0.82 for the full scale and construct-level values ranging from 0.79 to 0.93. Based on these satisfactory results, the finalized questionnaire was employed for large-scale data collection and subsequent empirical analysis. The final measurement items are presented in Table 1.

**Table 1:** Measurement items.

Source	Variables	Items	Code
Smink et al. (2020)	Perceived personalization (PP)	The platform provides a personalized fitting experience customized to each user’s individual characteristics.	PP1
		The platform provides a fitting experience perceived as highly personalized and unique to each user.	PP2
		The garments recommended by the platform are aligned with each user’s individual preferences and requirements.	PP3
		The platform’s recommendations align closely with each user’s individual style preferences.	PP4
Youn et al. (2023)	Perceived responsiveness (PR)	The platform’s interactive interface allows for swift modifications to garment combinations and overall outfit coordination.	PR1
		The platform demonstrates high responsiveness, enabling rapid, effective reactions to user inputs and interactions.	PR2
		The platform enables real-time, seamless interaction between users and the virtual fitting system.	PR3
Shelvia et al. (2020)	Convenience of artificial intelligence (CAI)	User interaction with the platform follows a clear intuitive process, making it easy to use.	CAI1
		Interaction with the platform requires minimal effort from users.	CAI2
		Using the platform’s services is straightforward and requires minimal effort from users.	CAI3
		Compared with traditional virtual fitting rooms, the platform provides a more convenient and efficient service experience.	CAI4
Shoukat et al. (2025)	Privacy concerns (PCs)	To what extent are you concerned that the platform could associate your facial or body data with your personal identity?	PC1
		To what extent are you concerned that the platform could collect and store sensitive information such as your body shape and measurements?	PC2
		To what extent are you concerned that your data could be used for commercial purposes unrelated to your personal use of the platform?	PC3
Lin and Kim (2016)	Intrusiveness concern (INC)	The platform collects or analyses an excessive amount of personal data, which makes me feel uncomfortable.	INC1
		The platform’s recommendations and prompts interfere with my normal shopping decision-making process.	INC2
		The platform has caused excessive disruption or interference during my use.	INC3
Nguyen et al. (2025)	Perceived behavioral control (PBC)	Using the platform’s services is entirely at my discretion.	PBC1
		You are able to bear the costs associated with using the platform’s services.	PBC2
		You possess sufficient knowledge to understand how to use the platform and its privacy policy.	PBC3
		You have the ability to manage and control personal data on this platform.	PBC4
Lin and Chuang (2018)	Attitude (AT)	Using this platform for virtual try-ons is enjoyable and appealing.	AT1
		You are willing to proactively explore and try out the platform’s new features or experiences.	AT2
		The platform’s virtual fitting experience does not leave me with a positive impression.	AT3
		Even if recommended by others, I would not be willing to use the platform.	AT4
Ruiz-Herrera et al. (2023)	Intention to use (IU)	You wish to use this platform to purchase clothing.	IU1
		Would you recommend other consumers to use this platform for online shopping?	IU2
		Would you intend to use this platform for future purchases?	IU3

### 3.3 Data collection

To ensure the sample was both representative and aligned with the research questions, an online questionnaire was used to collect data. The survey was conducted via the professional research platform Credamo (Wu et al., 2022), which allows for precise participant recruitment based on researcher-defined screening criteria. After the questionnaire was designed, it was made available to the target population between August 2025 and November 2025. The average completion time for the survey was approximately 8 min, and participants were incentivized with a nominal cash reward upon submitting their responses. The survey remained open for 4 months, ensuring extensive coverage of active users over different time periods and reducing the potential for sample bias related to any single timeframe. Additionally, responses that met any of the following criteria were excluded: (1) those completed in under 120 s, (2) incomplete responses, (3) responses terminated at the start of the survey, or (4) responses where identical answers were provided to all questions.

It should be noted that this study received ethical approval from the Research Ethics Review Committee of the School of Fashion at Zhejiang Sci-Tech University on 12 July 2025 (approval number: ZSTUFDE2025071208) and was conducted in strict accordance with the ethical principles outlined in the 1964 Declaration of Helsinki and its subsequent amendments. All interview participants provided written informed consent prior to participation. For the online questionnaire component—including both the pre-test and formal survey—an electronic informed consent procedure was implemented: participants were required to read the consent statement carefully and select the “Agree” option before continuing. All collected data were anonymized and used exclusively for academic research purposes.

Unlike previous studies that generalize findings to all online consumers, this research specifically targets Generation Z users aged 18–30. This demographic represents the primary user base of virtual fitting rooms (Trivedi, 2023) and digital retail platforms and is also the group most responsive to the influence of artificial intelligence (AI) technologies (Chan and Lee, 2023). The sample was restricted to the core cities of the Yangtze River Delta region, which not only constitutes one of China’s most dynamic areas for e-commerce development (Li, 2022) but also serves as a major innovation hub for digital retail and AI technologies, exemplified by companies such as Alibaba (Zhuo et al., 2021). Users in this region exhibit markedly higher levels of interest and engagement with AI technologies compared to those in other regions (Yang et al., 2024). This sampling strategy ensures strong alignment between the research participants and the study’s objectives.

A total of 750 responses were initially collected. After excluding invalid cases characterized by substantial missing data or completion times shorter than 120 s, 709 valid responses were retained, resulting in a validity rate of 94.5%. According to the “tenfold rule” proposed by Hair et al., the minimum sample size required for Partial Least Squares Structural Equation Modeling (PLS-SEM) should be at least 10 times the number of structural paths directed toward the most complex dependent variable (Hair et al., 2021). The final valid sample size of 709 in this study substantially exceeded this threshold, thereby ensuring the stability of path coefficient estimates and the robustness of the model analysis.

**Figure 2:**
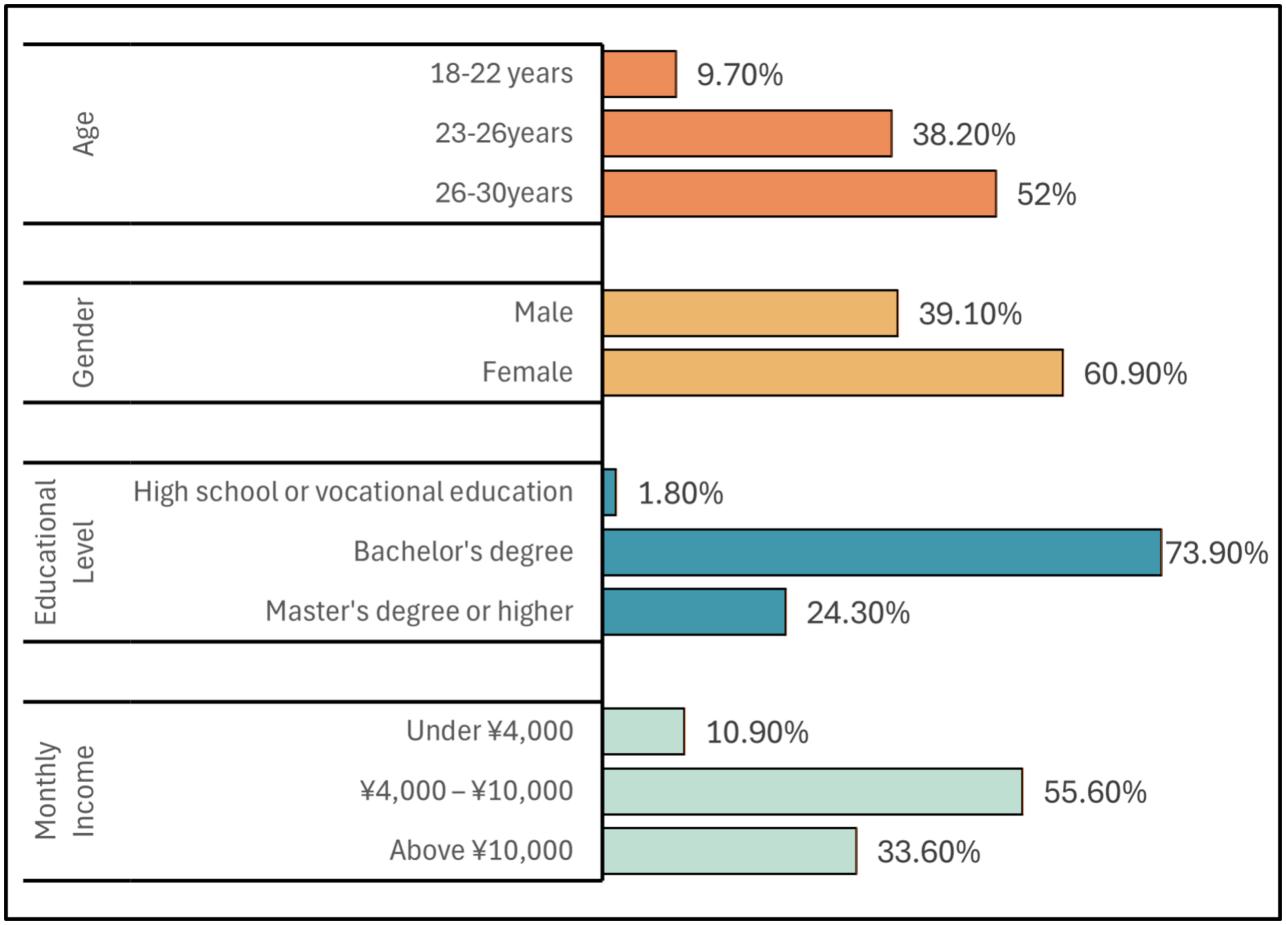
Sociodemographic features of respondents.

Regarding demographic characteristics, 39.1% of respondents were men and 60.9% were women. Regarding educational background, 73.9% of participants possessed a bachelor’s degree or higher, indicating a highly educated sample that aligns with Generation Z’s general profile of advanced educational attainment and strong technological adaptability (Espejo et al., 2024). The income distribution was primarily concentrated in the ¥4,000–10,000 range, encompassing 55.6% of respondents, which reflects this group’s substantial purchasing power. A summary of detailed demographic information is provided in Table 2 and Figure 2.

**Table 2:** Summary of the study sample’s characteristics.

Characteristics	Description	Number	Percentage (%)
Age	18–22 years old	69	9.7
	23–26 years old	271	38.2
	26–30 years old	369	52
Gender	Male	277	39.1
	Female	432	60.9
Education	Secondary education or vocational training	13	1.8
	Bachelor’s degree	524	73.9
	Master’s degree or higher	172	24.3
Income	Below ¥4,000	77	10.9
	¥4,000–10,000	394	55.6
	Above ¥10,000	238	33.6

Source: Authors’ contribution.

## 4 Data analysis based on PLS-SEM

### 4.1 Common method variance and multicollinearity assessment

To assess the potential presence of Common Method Variance (CMV), Harman’s single-factor test was conducted (Aguirre-Urreta and Hu, 2019). Using principal component analysis (PCA) in Statistical Package for the Social Sciences (SPSS) version 27.0, factors were extracted without rotation. The results indicated that the first extracted factor accounted for 19.372% of the total variance—well below the 50% critical threshold—demonstrating that no single factor dominated the variance explanation. This finding suggests that common method bias was not a major concern in this study. In addition, the Variance Inflation Factors (VIFs) for all constructs were below the critical value of 3, as shown in Table 3, thereby confirming the absence of multicollinearity among the variables.

**Table 3:** Reliability and validity analysis results.

Constructs	Coding	Mean	SD	Factor loading	Cronbach’s α	CR	AVE	VIF
Perceived personalization (PP)	PP1	5.74	0.653	0.722	0.723	0.825	0.544	1.367
	PP2	5.41	0.804	0.734				1.292
	PP3	5.70	0.790	0.658				1.389
	PP4	5.63	0.767	0.813				1.624
Perceived responsiveness (PR)	PR1	5.93	0.806	0.655	0.712	0.833	0.624	1.195
	PR2	5.96	0.953	0.780				1.700
	PR3	5.77	0.896	0.651				1.731
Convenience of artificial intelligence (CAI)	CAI1	5.95	0.651	0.818	0.716	0.819	0.533	1.388
	CAI2	5.99	0.705	0.814				1.428
	CAI3	5.85	0.682	0.846				1.230
	CAI4	6.00	0.667	0.784				1.444
Privacy concerns (PCs)	PC1	3.52	1.715	0.840	0.885	0.927	0.810	2.284
	PC2	3.12	1.754	0.681				2.740
	PC3	3.32	1.875	0.655				2.627
Intrusiveness concern (INC)	INC1	2.97	1.522	0.775	0.747	0.856	0.665	1.478
	INC2	2.46	0.983	0.719				1.653
	INC3	2.41	0.878	0.667				1.428
Perceived behavioral control (PBC)	PBC1	5.47	0.899	0.678	0.723	0.829	0.550	1.772
	PBC2	5.45	0.937	0.869				1.229
	PBC3	5.73	0.987	0.798				1.272
	PBC4	5.43	1.136	0.801				1.548
Attitude (AT)	AT1	6.06	0.654	0.770	0.713	0.823	0.538	1.398
	AT2	6.10	0.644	0.886				1.408
	AT3	5.84	0.650	0.932				1.272
	AT4	6.04	0.758	0.880				1.585
Intention to use (IU)	IU1	5.98	0.644	0.822	0.723	0.844	0.643	1.419
	IU2	5.73	0.899	0.807				1.427
	IU3	5.98	0.735	0.775				1.414

AVE, Average Variance Extracted; SD, standard deviation; VIF, Variance Inflation Factor.

### 4.2 Measurement model analysis

High levels of reliability and validity are essential indicators of the quality of measurement instruments. As presented in Table 3, both Cronbach’s *α* and Composite Reliability (CR) values for all constructs in the two experimental groups exceeded the recommended threshold of 0.70, indicating that each construct exhibited strong internal consistency and that the overall measurement scale demonstrated excellent reliability (Hair et al., 2011).

Before performing Structural Equation Modeling (SEM), an Exploratory Factor Analysis (EFA) was conducted to identify the underlying latent factor structure and assess the construct validity of the integrated model. The results revealed a Kaiser–Meyer–Olkin (KMO) value of 0.781 (>0.70), and Bartlett’s Test of Sphericity was statistically significant (*p* = 0.000 < 0.05), confirming the suitability of the data for factor analysis. Using varimax rotation, eight factors were extracted from the 28 items, collectively explaining 60.648% of the total variance. This suggests that the factor structure effectively represents the information contained in the original dataset. In addition, the minimum communalities value was 0.474 (>0.40), further supporting the adequacy of the factor solution. Overall, these results indicate that the questionnaire exhibits satisfactory construct validity.

A Confirmatory Factor Analysis (CFA) was subsequently performed to evaluate the model’s internal consistency, convergent validity, and discriminant validity. The analysis examined factor loadings, Composite Reliability (CR), and Average Variance Extracted (AVE). As presented in Table 3, all CR values exceeded 0.70, each construct’s AVE was above the recommended threshold of 0.50, and all standardized factor loadings were greater than 0.60 (Fornell and Larcker, 1981). These results collectively demonstrate that the measurement model exhibits satisfactory convergent validity.

Discriminant validity evaluates the extent to which each construct is distinct from the others in the measurement model. To assess discriminant validity, the model was examined using the Fornell–Larcker criterion (Wu et al., 2022). As presented in Table 4, the standardized correlation coefficients between all construct pairs were lower than the square root of their corresponding AVE values, indicating that all constructs demonstrated satisfactory discriminant validity.

**Table 4:** Discriminant validity assessment based on the Fornell–Larcker criterion.

Constructs	AT	CAI	INC	PBC	PP	PR	PC	IU
AT	0.734							
CAI	0.266	0.730						
INC	−0.393	0.023	0.815					
PBC	0.313	0.261	−0.451	0.742				
PP	0.192	0.266	−0.119	0.253	0.738			
PR	0.301	0.287	−0.250	0.075	0.138	0.790		
PC	−0.063	0.076	0.024	0.049	0.084	−0.059	0.900	
IU	0.445	0.114	−0.542	0.366	0.171	0.409	−0.016	0.802

### 4.3 Structural model analysis

This study employed three key metrics to evaluate the explanatory power, predictive capability, and overall model fit of the structural model, as shown in Table 5. According to established research guidelines, *R*^2^ represents the proportion of variance in the dependent variable explained by the model, with values of 0.19, 0.33, and 0.67 corresponding to weak, moderate, and strong explanatory power, respectively (Hair et al., 2011). The *R*^2^ values of the endogenous constructs indicated that the model explained 25.8% of the variance in Attitude (AT) (*R*^2^ = 0.258), 31.3% of the variance in Perceived Behavioral Control (PBC; *R*^2^ = 0.313), and 42.7% of the variance in Intention to Use (IU) (*R*^2^ = 0.427). These results suggest that the model’s endogenous latent variables exhibit moderate to strong explanatory power. Furthermore, Q^2^ was used as an indicator of the model’s predictive relevance. Using the Blindfolding algorithm, the study obtained Q^2^ values of 0.241, 0.295, and 0.368 for AT, PBC, and IU, respectively. Since all values exceeded zero, the model demonstrates adequate predictive validity. Finally, the overall model fit was assessed using the Standardized Root Mean Square Residual (SRMR), which yielded a value of 0.072—below the recommended cut-off of 0.08—indicating a satisfactory alignment between the model and the observed data.

**Table 5:** Structural model assessment and hypothesis testing results.

Construct	Path analysis	Hypotheses	*β*	*p*	Support	*R* ^2^	*Q* ^2^	SRMR
IU	PR → IU	H2b	0.268	0.000	Yes	0.427	0.368	0.072
	PC → IU	H4b	0.140	0.660	No			
	INC → IU	H5c	−0.328	0.000	Yes			
	AT→IU	H6	0.201	0.000	Yes			
	PBC → IU	H7	0.140	0.000	Yes			
AT	PP → AT	H1	0.079	0.034	Yes	0.258	0.241	
	PR → AT	H2a	0.134	0.000	Yes			
	CAI → AT	H3	0.220	0.000	Yes			
	PC → AT	H4a	−0.070	0.033	Yes			
	INC → AT	H5a	−0.354	0.000	Yes			
PBC	INC → PBC	H5b	−0.477	0.000	Yes	0.313	0.295	

**Figure 3:**
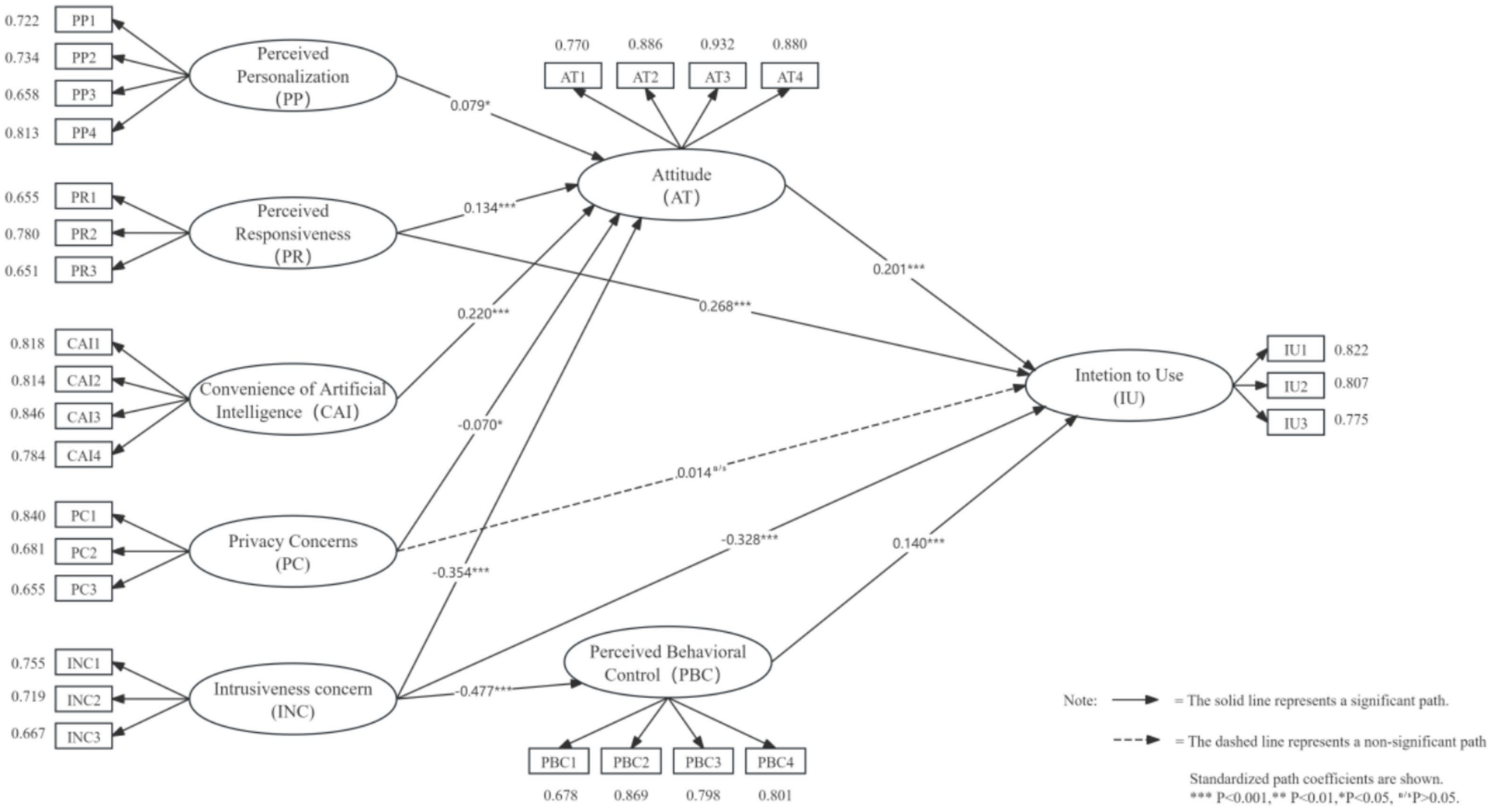
Structural model results.

The direct effects of the structural model are summarized in Table 5. As shown in Table 5 and Figure 3, the Intention to Use AI-VTO was significantly and positively influenced by Perceived Responsiveness (PR; *β* = 0.268, *p* < 0.001), Attitude (AT; *β* = 0.201, *p* < 0.001), and Perceived Behavioral Control (PBC; *β* = 0.140, *p* < 0.001), whereas Intrusiveness Concerns (INCs) exerted a significant negative effect (*β* = −0.328, *p* < 0.001). Accordingly, H2b, H6, H7, and H5c are supported. In contrast, Privacy Concerns (PC; *β* = 0.014, *p* > 0.05) had no significant effect on Intention to Use (IU); therefore, H4b is not supported.

Regarding the direct effects on Attitude (AT), the results indicated that Perceived Personalization (PP; *β* = 0.079, *p* < 0.05), Perceived Responsiveness (PR) (*β* = 0.134, *p* < 0.001), and Convenience of AI (CAI; *β* = 0.220, *p* < 0.001) had significant positive influences, while Privacy Concerns (PCs; *β* = −0.070, *p* < 0.05) and Intrusiveness Concerns (INCs; *β* = −0.354, *p* < 0.001) had significant negative effects. Accordingly, H1, H2a, H3, H4a, and H5a are supported.

Finally, Perceived Behavioral Control (PBC) was significantly and negatively influenced by Intrusiveness Concerns (INCs) (*β* = −0.477, *p* < 0.001), supporting H5b.

Overall, all hypotheses receive empirical support except for H4b.

### 4.4 Mediation effect analysis

This study employed the bootstrapping method recommended by MacKinnon et al., using 5,000 resamples to test the presence of mediating effects. This non-parametric approach does not assume a normal distribution of the sampling data. A mediation effect was considered significant when the 95% confidence interval (CI) did not include zero. The analysis primarily examined the mediating roles of Attitude (AT) and Perceived Behavioral Control (PBC) in the relationships among Perceived Personalization (PP), Perceived Responsiveness (PR), Convenience of AI (CAI), Privacy Concerns (PCs), Intrusiveness Concerns (INCs), and Intention to Use (IU).

The results of the mediation analysis are presented in Table 6. The 95% confidence intervals (CIs) for the indirect effects were as follows: PP → AT→IU (95% CI [0.001, 0.032]), PR → AT→IU (95% CI [0.012, 0.044]), CAI → AT→IU (95% CI [0.026, 0.067]), PC → AT→IU (95% CI [−0.029, −0.001]), and INC → AT→IU (95% CI [−0.100, −0.045]). As none of these intervals included zero, the results confirm that Perceived Personalization (PP), Perceived Responsiveness (PR), Convenience of AI (CAI), Privacy Concerns (PCs), and Intrusiveness Concerns (INCs) exert significant indirect effects on Intention to Use (IU) through the mediating role of Attitude (AT). These findings highlight that Attitude functions as a critical psychological mechanism linking users’ perceptions to their behavioral intentions toward AI-VTO technology.

**Table 6:** Results of mediating effect.

Mediating variable	Path	*β*	SD	*p*-value	CI 2.5%	CI 97.5%	Indirect effect or not
AT	PP → AT→IU	0.016	0.008	0.044	0.001	0.032	Yes
	PR → AT→IU	0.027	0.008	0.001	0.012	0.044	Yes
	CAI → AT→IU	0.044	0.010	0.000	0.026	0.067	Yes
	PC → AT→IU	−0.014	0.007	0.045	−0.029	−0.001	Yes
	INC → AT→IU	−0.071	0.014	0.000	−0.100	−0.045	Yes
PBC	INC → PBC → IU	−0.067	0.015	0.000	−0.099	−0.039	Yes

Furthermore, the regression-based mediation analysis produced the following result: INC → PBC → IU (95% CI [−0.099, −0.039]). Since the confidence interval did not include zero, the mediating effect was statistically significant. This finding indicates that Intrusiveness Concerns (INCs) significantly influence Intention to Use (IU) through the mediating role of Perceived Behavioral Control (PBC), highlighting the indirect pathway through which users’ perceptions of intrusiveness reduce their behavioral intentions toward AI-VTO adoption.

## 5 Multiple path analysis based on fsQCA

### 5.1 Variable selection and data calibration

The results of the PLS-SEM analysis indicate that Perceived Personalization (PP), Perceived Responsiveness (PR), Convenience of AI (CAI), Privacy Concerns (PCs), Intrusiveness Concerns (INCs), Perceived Behavioral Control (PBC), and Attitude (AT) exert significant influences on Intention to Use (IU). However, it remains unclear whether these factors exhibit configurational effects and which combinations of conditions most strongly shape users’ willingness to adopt AI-VTO. Therefore, this study employed these variables as antecedent conditions in a Fuzzy-set Qualitative Comparative Analysis (fsQCA) to further explore the causal configurations leading to high AI-VTO usage intention from a configurational perspective.

Before performing the fsQCA analysis, the questionnaire data were calibrated into fuzzy sets with membership scores ranging from 0 to 1 to improve the interpretability of the results. Since fsQCA is applicable only to unidimensional variables, and all antecedent constructs in this study consist of multiple measurement items, the arithmetic mean of each construct’s items was calculated prior to calibration. Following Ragin’s (2009) recommended procedure, three calibration anchors were established at the 5th percentile (full non-membership), the 95th percentile (full membership), and the 50th percentile (crossover point) (Ragin, 2009), as shown in Table 7.

**Table 7:** Calibration anchors for each variable.

Variable		Anchor Point		
		Full membership (membership = 0.95)	Crossover point (membership = 0.5)	Full non-membership (membership = 0.05)
Antecedent variable	Perceived personalization (PP)	6.50	5.75	4.75
	Perceived responsiveness (PR)	6.67	6.00	4.67
	Convenience of artificial intelligence (CAI)	6.75	5.75	5.25
	Privacy concerns (PCs)	6.33	2.67	1.33
	Intrusiveness concern (INC)	4.67	2.33	1.33
	Perceived behavioral control (PBC)	6.50	5.75	4.25
	Attitude (AT)	6.75	5.75	5.25
Outcome variable	Intention to use (IU)	6.67	6.00	4.67

### 5.2 Analysis of the necessity of a single condition

Before conducting the conditional configuration analysis, this study used fsQCA 4.0 software to perform a necessity analysis on each antecedent variable to determine whether any single factor constituted a necessary condition influencing AI-VTO usage intention. A condition was considered necessary when its consistency score exceeded 0.90 (Fiss, 2011). The results of the necessity condition analysis are presented in Table 8. None of the antecedent conditions surpassed the 0.90 consistency threshold, indicating that no individual variable in this study served as a necessary condition for influencing AI-VTO usage intention. Therefore, a configurational analysis of the antecedent variables was subsequently conducted to explore combinations of causal conditions associated with the outcome.

**Table 8:** Necessity and sufficiency tests of conditional variables.

Antecedent condition	Consistency		Coverage	
	High platform ambidexterity	Low platform ambidexterity	High platform ambidexterity	Low platform ambidexterity
PP	0.610336	0.566099	0.700971	0.577019
~PP	0.631711	0.706633	0.621277	0.616776
PR	0.737692	0.596814	0.726628	0.521726
~PR	0.514443	0.687286	0.589775	0.699283
CAI	0.707104	0.662117	0.681873	0.566659
~CAI	0.550623	0.628282	0.647415	0.655617
PC	0.646594	0.655398	0.672296	0.604784
~PC	0.619892	0.644870	0.669628	0.618239
INC	0.575540	0.771396	0.604476	0.719030
~INC	0.732477	0.575668	0.783093	0.546208
PBC	0.721801	0.543361	0.775520	0.518120
~PBC	0.551500	0.764587	0.576423	0.709233
AT	0.801692	0.633950	0.723617	0.507835
~AT	0.454731	0.654981	0.583288	0.745630

### 5.3 Configurational analysis of AI-VTO usage intention

This study incorporated seven antecedent variables: Perceived Personalization (PP), Perceived Responsiveness (PR), Perceived Convenience of Artificial Intelligence (CAI), Privacy Concerns (PCs), Intrusiveness Concerns (INCs), Perceived Behavioral Control (PBC), and Attitude (AT). A Fuzzy-set Qualitative Comparative Analysis (fsQCA) was conducted, with Generation Z’s willingness to use AI-VTO specified as the outcome variable. Given that the sample comprised 709 valid questionnaire responses, and following the conventional requirement that no fewer than 75% of cases be retained, the frequency threshold was set to 5, and the Proportional Reduction in Inconsistency (PRI) consistency threshold was set to 0.87 based on the case distribution in the truth table. The fsQCA 4.0 software produced three types of solutions—complex, parsimonious, and intermediate. Consistent with established reporting practice, this study focused on the intermediate solutions, supplemented by the parsimonious solutions for comparative reference. Table 9 presents the configurational analysis results for AI-VTO usage willingness, where configurations 2a and 2b, as well as 3a and 3b, represent two intermediate solutions derived from the same parsimonious solution, sharing identical core conditions.

**Table 9:** Results of configurational path analysis for AI-VTO usage intention.

Antecedent condition	AI-VTO usage intention				
	1	2a	2b	3a	3b
PP	⊗		⊙	⊙	⊙
PR	⊙	⊙	⊗	⊙	⊙
CAI	⊙	⊙		⊙	⊙
PC	X		⊙		X
INC		⊙	⊗	⊗	
PBC	⊗	⊗	●	●	●
AT	●	●	●	●	●
Raw coverage	0.265368	0.323883	0.249156	0.333014	0.274393
Unique coverage	0.0139764	0.047227	0.035966	0.0405981	0.00527114
Consistency	0.917526	0.888874	0.916381	0.939256	0.930486
Overall coverage	0.538961				
Overall consistency	0.871615				

● indicates the presence of a core condition, ⊙ indicates the presence of a peripheral condition, X indicates the absence of a core condition, and ⊗ indicates the absence of a peripheral condition.

Table 9 reports an overall consistency coefficient of 0.8716 and an overall coverage rate of 0.5390, indicating that the configurational paths demonstrate strong explanatory power. Five valid configurations associated with high AI-VTO usage intention were identified, each exhibiting a consistency value above 0.88, confirming that these pathways collectively account for the formation of usage intention. By categorizing the five configurations according to their core driving logic and conditional structures, three typical adoption patterns among Generation Z users can be summarized.

#### 5.3.1 Efficacy–trust type (configuration 1)

This pathway is valid primarily under low Privacy Concern conditions. Once a solid foundation of trust is established, high Perceived Responsiveness emerges as the main driver of usage intention. This reflects a rational balancing mechanism in which responsiveness serves as a core adoption criterion only within a secure and trustworthy environment.

#### 5.3.2 Experience-driven type (configurations 2a and 2b)

Centered on strong attitudinal commitment, this model highlights users’ prioritization of superior interactive experiences—embodied in Perceived Responsiveness, personalization, or AI-enabled convenience—while downplaying privacy or Intrusiveness Concerns. It represents an “experience-first” decision logic, where experiential gratification outweighs perceived risk.

#### 5.3.3 Control–convenience type (configurations 4 and 5)

Anchored in high Perceived Behavioral Control and typically accompanied by low Intrusiveness Concern, this model illustrates a more deliberate and cautious adoption orientation. Users seek both convenience and autonomy, embracing AI-VTO technologies only when they feel in control of operational processes and personal data. The underlying decision logic can be summarized as “comfortable adoption under control,” where technological benefits are accepted within clearly defined psychological and privacy boundaries.

## 6 Discussion

This study integrates the Privacy Calculus Model (PCM) and the Theory of Planned Behavior (TPB) to develop a comprehensive framework for explaining Generation Z consumers’ adoption intentions toward AI-VTO technology. Using a mixed-methods approach that combines PLS-SEM and fsQCA, this study not only examines the linear relationships and mediating pathways among variables but also identifies multiple configurational pathways leading to high usage intentions. The findings indicate that, when evaluating high-risk, high-reward technologies such as AI-VTO, Generation Z consumers’ decision-making processes exhibit pronounced characteristics of situational trade-offs and multi-path formation.

### 6.1 Interactive responsiveness and personalized value: the foundational drivers of attitude formation

At the level of the benefit structure, both Perceived Responsiveness (PR) and Perceived Personalization (PP) exert a significant positive effect on Attitude. Furthermore, PR exerts a direct positive effect on usage intention, which is consistent with findings reported in recent studies. This finding indicates that, in generative AI-driven virtual fitting contexts, users’ value judgments depend not only on outcome utility, but also on the quality of the interactive process and the degree of system–user alignment. First, responsiveness, as a critical characteristic of highly interactive digital services, reduces uncertainty and strengthens users’ perceptions of system capability and reliability, thereby promoting both favorable Attitude formation and usage intention. Related studies further confirm that the quality of real-time feedback and system responsiveness significantly influence users’ evaluations of their experience and their propensity to adopt AI systems (Chau et al., 2025; Hardcastle et al., 2025). Second, the positive effect of Perceived Personalization on Attitude is consistent with prior research on AI-based personalized services: when systems deliver content and feedback that more closely align with users’ needs and preferences, users are more likely to form favorable evaluations and demonstrate stronger willingness to engage (Timimi et al., 2025; Mani et al., 2025). Unlike traditional e-commerce recommendation systems, which emphasize one-time output performance, AI-VTO constitutes a continuous interactive process characterized by feedback loops and dynamic matching. Consequently, interaction fluency and perceived fit more effectively explain the formation of Attitudes and adoption intentions (Alghaswyneh, 2025). Overall, PR and PP jointly constitute the principal sources of perceived benefit in AI-VTO: the former enhances the interaction experience and directly increases usage intention, whereas the latter improves perceived relevance and fit, primarily exerting its influence through Attitude.

### 6.2 The mechanism of perceived AI convenience in attitude formation

The significant positive effect of Convenience of Artificial Intelligence (CAI) on Attitude indicates that, within generative AI-driven interactive environments, users’ perceptions of usability and cognitive load remain critical evaluation criteria, which is consistent with recent research findings. When systems enable users to complete complex tasks in an intuitive and low-complexity manner, users are more likely to develop positive Attitudes, because reduced cognitive load enhances comfort and perceived control during use (Huang and Rust, 2021; Wirtz et al., 2018). Unlike traditional online shopping environments, generative AI systems often involve greater algorithmic complexity and more opaque decision-making processes. Research indicates that when users struggle to understand a system’s operational logic, heightened perceptions of complexity and technological distance reduce their overall evaluations of the system (Shin, 2021). Consequently, in the AI-VTO context, convenience extends beyond operational efficiency to encompass the platform’s ability to manage or mitigate perceived technical complexity through effective interaction design. This includes enhancing usability through automated matching, clear process flows, and reduced interface burden, thereby improving users’ Attitudes toward the technology (Liao and Sundar, 2022). Furthermore, the inherent uncertainty of generative AI outputs may trigger user anxiety and reduce reliance on the technology when such outputs are perceived as unpredictable or lacking transparency (Kellogg et al., 2020; Longoni and Cian, 2022). High levels of convenience and clear interaction pathways can partially alleviate resistance arising from such uncertainty, thereby further strengthening positive evaluations of and Attitudes toward AI-VTO.

### 6.3 The dual-path structure of risk perception: differential effects of intrusiveness and privacy concerns

The findings reveal a clear structural differentiation across risk dimensions: Intrusiveness Concerns (INCs) exert a significant negative effect on Intention to Use (IU), substantially weakening both Attitude (AT) and Perceived Behavioral Control (PBC). In contrast, Privacy Concerns (PCs) do not exert a significant direct effect on IU but have a significant negative effect on AT. These findings suggest that, within the AI-VTO context, users do not perceive privacy risk as a single, homogeneous psychological construct. Instead, users differentiate at least two distinct forms of risk perception: (1) Privacy Concerns, which are primarily cognitive evaluations reflecting general worries about information misuse, leakage, and secondary use; and (2) intrusive feelings, which are experiential and affective reactions characterized by immediate discomfort arising from excessive tracking, boundary-crossing data utilization, or perceived disturbance. The asymmetry in both the intensity and influence pathways of these two risk types highlights the nuanced structure of risk mechanisms within AI-VTO environments.

#### 6.3.1 Direct inhibitory effects of experiential risks

The direct negative effect of Intrusiveness Concerns on willingness to use is consistent with recent findings that intrusiveness is a key trigger of user rejection of personalized services. As AI personalization and targeting mechanisms increasingly rely on sensitive personal data, users are more likely to interpret highly targeted content delivery as overstepping personal boundaries or constituting excessive intervention, thereby inducing avoidance and resistance. Recent reviews of personalized advertising research indicate that although personalization generally enhances Attitudes and persuasiveness, its effectiveness is significantly reduced and user resistance is triggered when it is perceived as intrusive or uncomfortable (Yeo et al., 2025). In studies of augmented reality and virtual fitting rooms, privacy- and risk-related variables have also been shown to undermine key psychological processes and subsequent behavioral intentions during immersive experiences. For example, recent research on AR-based virtual fitting indicates that Intrusiveness Concerns weaken the positive effects of immersive experiences and cognitive engagement on purchase intention (Lavoye and Kumar, 2025). Collectively, this body of evidence suggests that, in highly personalized and interactive AI-VTO contexts, intrusiveness is more readily perceived by users as a negative experiential cue, thereby directly reducing their willingness to engage.

#### 6.3.2 Evaluation pathways for cognitive risks

This study found that Privacy Concerns had no significant effect on usage intention but exerted a significant negative effect on Attitude. This finding differs from that of some earlier studies, which suggested that Privacy Concerns directly inhibit behavioral intention. According to the traditional privacy calculus framework, when individuals weigh perceived risks against benefits, heightened Privacy Concerns should reduce their willingness to disclose sensitive information or adopt new technologies. However, recent studies across diverse digital service contexts have consistently reported weak or inconsistent relationships between Privacy Concerns and actual behavior (Hernández et al., 2025). Systematic reviews further indicate that the predictive power of Privacy Concerns is highly context-dependent; in emerging technology settings such as AI, the Internet of Things, and augmented reality, data sensitivity, risk evaluation, and contextual cues reshape the relationship between Privacy Concerns and behavior. As a result, traditional privacy models may be insufficient to reliably explain micro-level decision-making mechanisms (Herriger et al., 2025).

Against this backdrop, privacy calculus theory emphasizes that users are not solely driven by Privacy Concerns, but instead evaluate benefits and convenience within a broader risk–reward trade-off. When anticipated benefits significantly outweigh perceived risks in a specific context, the negative effect of Privacy Concerns on behavioral intention may be weakened or even become insignificant (Kezer et al., 2022). Therefore, the finding that Privacy Concerns did not directly inhibit usage intention in this study does not imply that privacy-related factors are unimportant. Rather, it likely reflects a shift in the pathway through which Privacy Concerns exert influence: in highly interactive, high-immediacy feedback scenarios such as AI-VTO, where benefits are readily observable, user decisions are more strongly driven by perceived gains such as convenience, interactive experience, and matching accuracy. In such contexts, Privacy Concerns primarily influence usage intention indirectly through Attitude as an evaluative mediator. Overall, these findings suggest that the mechanisms through which Privacy Concerns influence behavior in generative AI environments are characterized by greater contextual specificity and multiple pathways, thereby providing empirical support for extending the privacy paradox to emerging technological contexts.

### 6.4 Mediating mechanisms of perceived behavioral control

The findings indicate that Perceived Behavioral Control (PBC) exerts a significant positive effect on intention to use (IU), whereas intrusive concerns (INC) exert a significant negative effect on PBC. This relationship highlights the central role of perceived control in adoption decisions involving data-intensive generative artificial intelligence systems. Perceptions of intrusiveness not only weaken individuals’ overall evaluative Attitudes but also further inhibit adoption intention by reducing their subjective sense of behavioral controllability.

According to the Theory of Planned Behavior (TPB), Perceived Behavioral Control refers to an individual’s subjective assessment of their ability and available resources to perform a specific behavior. This perception can directly influence behavioral intention while also indirectly shaping confidence in decision-making. In the AI-VTO context, which relies heavily on personal data input and algorithmic processing, Perceived Behavioral Control extends beyond assessments of operational capability to encompass individuals’ sense of control over data collection boundaries, authorization mechanisms, and privacy settings. When systems are perceived as excessively intrusive or as overstepping boundaries in the use of personal information, individuals are more likely to develop a sense of losing control over personal boundaries, thereby reducing their ability in Perceived Behavioral Control (Shin et al., 2022).

Therefore, in this context, “control” manifests not only as the technical ability to operate the system, but also as users’ subjective assessment of whether they can regulate how their personal data are used and processed during interaction. An increased sense of intrusiveness reinforces perceptions that the system is uncontrollable, thereby diminishing Perceived Behavioral Control; conversely, reduced perceived control further suppresses Intention to Use. This mechanism suggests that, within generative AI-driven interactive services, risk influences behavioral intention not only through attitudinal pathways but also through a deeper structural mechanism that erodes perceived control (Aysolmaz et al., 2023).

Existing research on e-commerce and digital services has shown that, in online environments, a sense of control is closely associated with risk management capability, control over information disclosure, and perceptions of transaction security. It therefore plays a pivotal role in shaping behavioral intention (Rodríguez-Priego et al., 2023; Jadil et al., 2022). In highly data-dependent scenarios such as AI-VTO, this sense of control is not merely a matter of operational capability, but also constitutes a vital component of boundary governance and trust formation. When platforms are perceived as intrusive, individuals not only develop negative affective evaluations but are also more likely to experience a cognitive sense of “loss of control.” This, in turn, further suppresses adoption intention at the decision-making level.

### 6.5 Three adoption logics from a configurational perspective

Based on the fsQCA results, this study identified three typical pathways leading to high usage intention. Unlike linear models, which emphasize the net effect of a single variable, configurational analysis suggests that high adoption intention is typically shaped by multiple conditions acting in combination, rather than by any single factor in isolation (Bozkir and Kasneci, 2025). Existing fsQCA research further suggests that, in the context of emerging digital technology adoption, outcomes often exhibit equifinality, meaning that different combinations of conditions can lead to the same result (Porfírio et al., 2024).

From a broader perspective of emerging technology research, this multi-path coexistence structure is not unique to AI-VTO. A systematic review of privacy behaviors within AI, augmented reality, IoT, and big data contexts reveals that user decisions exhibit pronounced situational dependency. Risks, benefits, and control cues interact differently across technological scenarios, jointly influencing adoption outcomes (Herriger et al., 2025). Consequently, within AI-VTO contexts involving bodily imagery, dimensional information, and hyper-personalized recommendations, Generation Z’s high adoption propensity is also more likely to manifest through multiple coexisting pathways rather than a singular mechanism (Bunea et al., 2024).

Based on the combinations of core conditions characterizing each pathway, this study identifies three configurations leading to high usage intention: the low Privacy Concern–high responsiveness pathway, the high Attitude-dominance pathway, and the high control–low intrusiveness pathway.

#### 6.5.1 Low privacy concerns—high responsiveness pathway

The first pathway is characterized by the combination of low Privacy Concerns and high Perceived Responsiveness. This finding suggests that, for some Generation Z users, a low perception of risk is a prerequisite for adoption, whereas the system’s ability to provide high-quality feedback and efficient interaction further determines whether they develop a strong Intention to Use it.

This finding broadly aligns with the central conclusion of existing privacy research that users are generally reluctant to adopt new technologies under conditions of perceived high risk. Relevant studies indicate that Privacy Concerns often serve as an initial “boundary filter”; only when users perceive risks to be acceptable do benefit-related factors become more likely to influence subsequent decision-making (Bol et al., 2018). Therefore, this pathway not only illustrates that low risk facilitates adoption, but also demonstrates that, in highly data-dependent scenarios such as AI-VTO, risk may first operate as a threshold filter, after which high responsiveness drives adoption beyond this initial barrier.

From the perspective of Generation Z’s characteristics, this pathway also appears to be practically meaningful. Research on young consumers in AI retail contexts indicates that, although this demographic is generally open to AI, it remains highly sensitive to trust-related issues and vigilant about risks associated with data use (ElSayad and Mamdouh, 2024). Consequently, for some Generation Z users, system responsiveness and interaction quality are more likely to translate into genuine adoption motivation only when Privacy Concerns remain low.

Overall, this pathway primarily complements existing privacy calculus research. It demonstrates that, in highly interactive scenarios such as AI-VTO, risks and benefits do not necessarily enter the decision-making process in parallel. Instead, they may follow a phased structure characterized by “risk screening first, followed by response-driven action.”

#### 6.5.2 High-attitude-dominance pathway

The second pathway is centered on a high Attitude as its core condition. This pathway suggests that, once users have formed sufficiently positive overall evaluations, the inhibitory effect of risk-related variables is markedly reduced, even if such risks have not disappeared entirely. In other words, high adoption intention arises not because risks are absent, but because benefit cues have been incorporated into a strong positive Attitude. This overall evaluative structure then drives adoption.

This finding is consistent with certain conclusions in the privacy paradox literature. Existing literature indicates that, in scenarios characterized by high returns, strong feedback, and high experiential value, users may express some degree of Privacy Concern without necessarily abandoning technology use outright; rather, they are more likely to continue using the technology when their overall evaluation remains predominantly positive (Khaksar et al., 2024). Building on this, the present study further demonstrates that, in scenarios such as AI-VTO, which emphasize visual feedback, personalized matching, and continuous interaction, positive Attitudes may serve as a crucial mediating mechanism that reduces the weight of perceived risks.

This finding also complements existing linear research. Previous studies have often treated Privacy Concerns as relatively stable negative predictors; however, the configurational results of this study indicate that the inhibitory effect of risk is highly conditional, with its influence mitigated by the formation of positive Attitudes. Research on Generation Z’s AI-assisted online shopping behavior similarly indicates that perceived usefulness, positive experiences, and favorable overall evaluations significantly enhance their propensity to purchase or use such services (Dewalska-Opitek et al., 2024). Consequently, this pathway suggests that the privacy paradox does not necessarily signify a failure of risk perception, but rather that positive Attitudes may provide phased compensation for perceived risk in specific high-reward scenarios.

Overall, this pathway broadly supports the central conclusion of privacy paradox research that perceived benefits may offset perceived risks. However, it further indicates that such compensation is not universal, but is more likely to occur in highly interactive, highly responsive, and highly personalized contexts such as AI-VTO.

#### 6.5.3 High control—low intrusiveness pathway

The third pathway is characterized by high behavioral control, typically accompanied by low concerns about intrusiveness. This pathway suggests that, for another segment of Gen Z users, the controllability of the technology and its ability to avoid overstepping personal boundaries are more important than high experiential value or responsiveness alone. When users believe they can manage the interaction process, understand how their data are used, and avoid excessive system intervention, the benefits of convenience and efficiency are more readily translated into a higher Intention to Use.

This finding broadly aligns with existing research on the relationship between perceived control and technology adoption. Extensive research on technology adoption demonstrates that perceived control significantly influences users’ behavioral intentions and is particularly critical in highly complex and uncertain technological environments (Wei N. et al., 2025). Similarly, research on generative artificial intelligence and emerging technology use indicates that users’ assessments of system transparency, comprehensibility, and boundary management substantially affect their trust and subsequent Intention to Use such technologies (Huang et al., 2025).

This pathway also complements existing privacy research. Traditional studies often emphasize the importance of risk reduction for adoption. In contrast, this study demonstrates that, under certain conditions, enhancing perceived control itself can integrate perceived risk and thereby stabilize adoption (Hansen et al., 2018). In other words, users do not necessarily demand the complete elimination of risk, but they do require that risks remain within a scope they can understand and manage. For Generation Z, this logic of “acceptable if controllable” is particularly salient. Although this demographic information exhibits high engagement with digital technologies, it also demonstrates growing concern about transparency, fairness, data privacy, usage boundaries, and autonomy in AI use (Dewalska-Opitek et al., 2024).

Consequently, this pathway further extends the implications of Perceived Behavioral Control in AI-VTO contexts. It demonstrates that a sense of control extends beyond operational capability to encompass users’ subjective assessment of whether the interaction process and data boundaries remain manageable.

#### 6.5.4 Comparative interpretation of the three pathways

Overall, although all three pathways lead to high adoption intention, their underlying logics differ. Existing configurational research indicates that, in emerging technology adoption contexts, different combinations of conditions can yield the same outcome. This suggests that high adoption intention often reflects equifinality rather than a single-path structure. This indicates that Generation Z’s high adoption intention toward AI-VTO does not have a single optimal explanation, but instead is likely to stem from diverse psychological foundations and decision-making structures (Dewalska-Opitek et al., 2024).

More specifically, the first pathway reflects a risk-threshold logic. In this pathway, users do not disregard privacy risks; rather, they first assess whether such risks fall within an acceptable range. Only when Privacy Concerns are relatively low do system responsiveness and interaction quality translate into adoption motivation (Pentina et al., 2016). The second pathway reflects a benefit-compensation logic. In this pathway, although risk factors remain present, users’ strong positive Attitudes allow personalized benefits, visual feedback, and interactive experiences to progressively offset perceived risks, thereby fostering high adoption intention (Khaksar et al., 2024). The third pathway reflects a control-first logic. In this pathway, users do not demand the complete elimination of risk, but instead prioritize their ability to understand and manage data usage processes, control the pace of interaction, and avoid excessive system intervention. When this sense of control is sufficiently strong, the benefits of convenience and efficiency are more readily translated into stable adoption intention (Huang and Liu, 2025).

In relation to the existing literature, these three pathways further complement current research. Previous studies on privacy calculus and technology adoption have typically favored a single-path explanation of behavioral intention, assuming that risks and benefits exert relatively stable linear effects on adoption. However, a growing body of research on AI and emerging technologies indicates that users’ evaluations of technology are inherently context-dependent. Benefits, risks, and cues of control interact in distinct ways across different scenarios to shape usage judgements (Dorotic et al., 2024). Consequently, in highly interactive, personalized, and data-dependent contexts such as AI-VTO, the same level of usage intention may rest on different risk–benefit configurations and psychological assessments.

From the perspective of the privacy paradox, these three pathways also carry distinct implications. The low Privacy Concern–high responsiveness pathway aligns more closely with efficacy-driven decision-making under conditions of acceptable risk. Its core logic involves first confirming that risks are low and then determining adoption based on system performance; as such, it does not constitute a typical privacy paradox. The high control–low intrusiveness pathway reflects a robust adoption logic grounded in controllability. Users do not disregard risks; rather, they continue using the technology after confirming their ability to manage data use and interaction processes; thus, this pathway does not fit the typical “concern–behavior inconsistency” pattern. By contrast, the Attitude-dominant pathway most closely embodies the characteristics of the privacy paradox. In this pathway, risk factors are not entirely absent, yet users maintain a high adoption intention due to an overall positive evaluation. Relevant research further indicates that the privacy paradox is not a universally stable phenomenon, but is more likely to emerge in specific high-reward, high-convenience, or high-experience contexts (Connolly et al., 2025).

This comparative analysis further demonstrates that Generation Z should not be simplistically characterized as a monolithic group that is uniformly privacy-conscious and technology-dependent. Rather, they are better understood as a user group that adopts distinct decision-making logics across different contexts: some prioritize assessing whether risks are acceptable; others are more inclined to downplay risk under conditions of high experiential value; while still others rely more heavily on a sense of control to integrate risk perceptions. Research on Generation Z’s AI adoption similarly indicates that, although this demographic maintains an overall open Attitude toward AI, their specific adoption decisions remain significantly influenced by the combined effects of trust, perceived security, perceived utility, and contextual factors (Mustafa et al., 2026).

Collectively, these three pathways demonstrate that Generation Z’s adoption decisions in highly data-dependent, interactive technological contexts such as AI-VTO exhibit strong contextual specificity. Their high adoption intention is not driven by any single factor, but emerges from the interplay of multiple psychological mechanisms, including risk perception, overall Attitude, and perceived control. Consequently, understanding Generation Z’s adoption behavior in AI-VTO contexts requires moving beyond explanations based solely on the net effects of individual variables. Instead, attention should be directed toward the multifaceted trade-off logics associated with different combinations of conditions.

### 6.6 Theoretical integration and contributions

The theoretical contribution of this study lies not only in applying the Theory of Planned Behavior (TPB) and the Privacy Calculus Model (PCM) to the context of AI-driven virtual try-on (AI-VTO), but also in deepening their integration and refining the structural logic and boundaries of applicability of these two frameworks within generative AI environments. Unlike prior research, which has predominantly explained technology adoption through a single theoretical framework, this study emphasizes that, within highly data-dependent and interactive AI service contexts, a distinct psychological pathway exists between risk–benefit trade-offs and the formation of behavioral intention. This pathway can only be adequately explained through the integration of multiple theoretical perspectives.

#### 6.6.1 From risk–benefit trade-offs to intent formation: clarifying the theoretical integration mechanism

Privacy calculus theory posits that individuals rationally weigh perceived risks against perceived benefits in digital contexts. However, existing research often treats risks and benefits as direct antecedents of behavioral intention without sufficiently explaining how they are psychologically integrated and translated into an intention structure. By contrast, the Theory of Planned Behavior (TPB) offers a clear framework for intention formation by positing that Attitude and Perceived Behavioral Control are key determinants of behavioral intention.

Building on this, the present study proposes and empirically validates an integration mechanism whereby risk–benefit evaluations enter the intention structure through an “Attitude–control perception” pathway. Risks and benefits do not directly determine adoption; instead, they indirectly shape usage intention by influencing overall Attitude (AT) and Perceived Behavioral Control (PBC). This integrative logic addresses recent critiques concerning the inadequacy of trade-off-to-translation mechanisms in privacy decision-making and provides a structured pathway for explaining the complex relationship between risk and behavior in highly interactive AI contexts (Pathak and Bansal, 2024).

Accordingly, this study clarifies the functional division between the Privacy Calculus Model (PCM) and the Theory of Planned Behavior (TPB): the PCM provides the “trade-off content,” whereas the TPB provides the “translation structure.” Together, these two frameworks constitute a complete explanatory mechanism for integrating risks and benefits into behavioral intention.

#### 6.6.2 Revision of privacy computing theory: structural differentiation of risk pathways

Privacy calculus theory typically assumes that risk exerts a linear negative effect on behavioral intention. However, our findings reveal that risk structures exhibit differentiated psychological pathways: concerns about intrusiveness exert a direct inhibitory effect on behavioral intention, whereas Privacy Concerns primarily influence behavioral intention indirectly through Attitude. This finding is consistent with recent research on the privacy paradox in AI contexts, which suggests that risk perception does not necessarily lead to behavioral inhibition; rather, its impact depends on the type of risk and the situational context. Experiential risks, such as perceived intrusiveness, are more likely to trigger immediate emotional responses and avoidance tendencies, whereas cognitive Privacy Concerns tend to enter decision-making structures through rational evaluation.

Consequently, this study revises the assumption of risk homogenization within the Privacy Calculus Model (PCM) by proposing that experiential and cognitive risks should be distinguished in terms of their causal pathways within highly interactive generative AI services (Masur and Ranzini, 2025). This refinement helps explain why Privacy Concerns exert limited influence on behavioral intention in certain AI applications, thereby advancing privacy calculus theory toward a more fine-grained mechanistic account.

#### 6.6.3 Contextual and configurational explanations of the privacy paradox

Existing privacy paradox research has predominantly explained the discrepancy between concerns and behavior through the lens of net variable effects. However, this study employs fsQCA to identify three adoption pathways, revealing that the privacy paradox primarily manifests in the Attitude-dominant pathway. By contrast, it is not clearly evident in either the low Privacy Concern–high responsiveness pathway or the high control–low intrusiveness pathway.

This finding indicates that the privacy paradox is not a stable psychological trait of Generation Z, but rather a contextual outcome that arises under specific risk–benefit structures. Related research on AI adoption also suggests that personalized benefits and convenience may compensate for perceived risks in certain contexts. Consequently, this study reconceptualizes the privacy paradox from a single-variable perspective of behavioral inconsistency to a decision pattern shaped by conditional configurations, thereby providing a more explanatory framework for understanding paradoxical phenomena across diverse AI application contexts.

Thus, this research not only enriches consumer behavior theory in the context of generative AI but also offers a transferable theoretical framework for understanding adoption mechanisms in augmented reality retail, intelligent recommendation systems, and other data-intensive AI services.

### 6.7 Management insights

From a design philosophy perspective, this study advocates the principle of humble interaction, which emphasizes reducing perceived intrusiveness as a central objective of user experience design. In AI-VTO contexts, this requires minimizing unnecessary pop-ups and prompts, ensuring that recommendations appear naturally and at appropriate moments, and giving users greater control over the pace of interaction. In this sense, technology should support users in a subtle and unobtrusive manner rather than imposing itself forcefully.

In addition, platforms should develop convenience-oriented experience optimization strategies by treating convenience as a core dimension of the overall user experience. The goal is to create an experience that feels effortless, low-burden, and time-saving, while allowing complex algorithmic processing and feedback to occur almost imperceptibly. Such a design not only improves the immediate interaction experience but also reinforces positive Attitudes toward the system, thereby indirectly enhancing usage intention.

The findings further suggest the importance of dynamic and segmented user engagement strategies. Because different users may arrive at high usage intention through different psychological pathways, platform design should be tailored accordingly. For users in the low Privacy Concern–high responsiveness pathway, emphasis should be placed on optimizing system responsiveness and feedback quality while maintaining trust through transparent security assurances. For users in the Attitude-dominant pathway, platforms should continuously strengthen rendering quality, personalized recommendations, and interactive experience in order to reinforce favorable overall evaluations. For users in the high Perceived Behavioral Control–low intrusiveness pathway, clear data dashboards, preference management functions, and algorithmic explanations should be provided to enhance their sense of control and reduce perceived intrusiveness.

The study also indicates that the privacy communication narrative should be redesigned. Rather than functioning merely as legal disclaimers, privacy policies should be reframed as part of the user experience. Accordingly, communication should move beyond emphasizing how data are protected and instead clarify how data-use boundaries are defined to prevent intrusive experiences and support a more comfortable and autonomous fitting process. This shift may help reduce users’ perceptions of boundary violation and strengthen trust in the platform.

Finally, Perceived Behavioral Control should be operationalized as a core functional module in platform design. This may include features that allow users to manage their fitting history autonomously, calibrate or reset recommendation preferences with one click, and adjust the factors influencing algorithmic recommendations through visualized controls. By embedding control directly into product functions, platforms can transform users from passive data providers into active participants in the service experience, thereby supporting more stable and sustainable adoption.

### 6.8 Research limitations and future directions

#### 6.8.1 Research limitations

Despite systematic efforts toward theoretical integration and methodological innovation, this study retains several limitations that warrant formal acknowledgement.

First, with respect to the research design, this study employs cross-sectional survey data analyzes them using PLS-SEM and fsQCA. Although multi-method integration enhances the robustness and interpretive depth of the findings, cross-sectional data are inherently limited in their ability to reveal dynamic causal relationships among variables. Particularly in AI contexts, users’ perceptions of privacy risks and functional benefits may evolve over time as usage experience accumulates, technological iterations occur, or external events unfold. Consequently, the risk–benefit integration mechanism identified here primarily reflects psychological structures at a specific point in time rather than long-term developmental processes.

Second, regarding sample and contextual boundaries, this study focuses on Generation Z consumers in China’s Yangtze River Delta region. Although this group is highly representative within digital environments, their privacy perceptions and technology adoption logic may be shaped by specific institutional environments, regulatory frameworks, and cultural values. Consequently, the generalizability of these findings to different countries or institutional contexts requires further empirical validation.

Finally, regarding measurement, this study primarily relies on self-report scales to assess Privacy Concerns, Intrusiveness Concerns, and usage intentions. Although these scales demonstrate satisfactory reliability and validity, self-reported data may nevertheless be subject to social desirability bias, *post hoc* rationalization, and cognitive consistency tendencies. This limitation may constrain the accurate reflection of actual behavioral outcomes.

#### 6.8.2 Future research directions

In light of the aforementioned limitations, future research may proceed along several directions. First, with respect to theoretical expansion, future studies could incorporate perspectives from behavioral economics and cognitive psychology to provide a more systematic explanation of the privacy paradox. Compared with rational deliberation models, cognitive biases and heuristic processing mechanisms may play a substantial role in privacy decision-making processes. Integrating these mechanisms into analytical frameworks could advance privacy decision research from static structural models toward more dynamic cognitive integration models.

Second, comparative studies across cultural and institutional contexts would offer substantial value. Diverse digital governance environments and social trust structures may influence risk–benefit weighting processes, thereby altering the manifestations of the privacy paradox. Future research could further examine the boundary conditions of this study’s model using multi-contextual samples.

Overall, this study provides a structured explanation of Gen Z’s privacy trade-off mechanisms in AI-driven virtual fitting contexts. Nevertheless, the complexity of privacy decision-making warrants continued investigation. Future research integrating methodological innovation and theoretical refinement will contribute to the development of more dynamic, contextualized, and multi-layered explanatory frameworks.

## 7 Conclusion

This study systematically examined Generation Z consumers’ adoption of AI-VTO technology by integrating the Privacy Calculus Model (PCM) with the Theory of Planned Behavior (TPB) and employing a hybrid methodological approach combining PLS-SEM and fsQCA. The main conclusions are as follows.

First, Generation Z’s adoption of AI-VTO reflects a complex and multidimensional process of balancing risks and benefits within specific contexts. The findings indicate that concerns about intrusiveness exert a more immediate inhibitory effect on usage intention than traditional Privacy Concerns, whereas Perceived Behavioral Control and positive Attitudes serve as two core drivers of adoption.

Second, one of the study’s most important findings is the identification of three equifinal yet logically heterogeneous pathways leading to high adoption intention among Generation Z users: the low Privacy Concern–high responsiveness pathway, the high control–low intrusiveness pathway, and the Attitude-dominant pathway. This finding indicates that there is no single “optimal pathway” to technology adoption.

Finally, these findings offer an important refinement and extension of privacy paradox theory. The study demonstrates that the privacy paradox is not a universal characteristic of all Generation Z users, but is more concentrated in the Attitude-dominant pathway. By contrast, the adoption logic of other users is more strongly shaped by trust formation and the maintenance of perceived control. Research on Generation Z further suggests that this cohort is not composed merely of “privacy-indifferent” digital natives, but rather of users who strategically balance personalized benefits against privacy protection (Willems et al., 2023). Consequently, Generation Z should not be understood as a monolithic group that is simultaneously privacy-concerned and technology-dependent; rather, they should be recognized as contextual decision-makers who employ diverse strategies to manage privacy risks across different digital scenarios.

From a practical perspective, this study suggests that, while pursuing technological benefits, industry actors must respect users’ psychological boundaries and decision-making autonomy. Firms should respond flexibly to the core needs of different user groups through three mutually reinforcing strategies: building foundational trust, empowering users with control, and creating high-quality experiences. Only in this way can the healthy and sustainable development of AI-VTO technology be achieved.

## Data Availability

The raw data supporting the conclusions of this article will be made available by the authors, without undue reservation.
